# Specific situation theory of the transition experience lived by people with intestinal ostomies and their families*

**DOI:** 10.1590/1518-8345.7979.4858

**Published:** 2026-06-15

**Authors:** Angélica Dalmolin, Eduardo da Silva Gomes, Juliano Teixeira Moraes, Leonardo Rozinelli, Maria Ribeiro Lacerda, Nara Marilene Oliveira Girardon-Perlini

**Affiliations:** 1 Universidade Federal de Santa Maria, Enfermagem, Santa Maria, RS, Brazil.; 2 Scholarship holder at the Coordenação de Aperfeiçoamento de Pessoal de Nível Superior (CAPES), Brazil.; 3 Scholarship holder at the Conselho Nacional de Desenvolvimento Científico e Tecnológico (CNPq), Brazil.; 4 Universidade Federal de São João del-Rei, Enfermagem, Minas Gerais, MG, Brazil.; 5 Universidade Federal de Santa Maria, Medicina, Santa Maria, RS, Brazil.; 6 Universidade Federal do Paraná, Enfermagem, Curitiba, PR, Brazil.

**Keywords:** Ostomy, Family, Nursing Care, Nursing Theory, Enterostomal Therapy, Nursing Models

## Abstract

**Objective:**

to develop a specific situational theory representative of the transition experience of people with intestinal ostomies and their families, based on Afaf Meleis’ Theory of Transitions.

**Method:**

theoretical study, guided by the theory-research-theory strategy and based on an integrative approach, which combines deductive and inductive strategies. In the deductive stage, the Theory of Transitions was listed to provide a conceptual structure and organize theoretical thinking. Inductive strategies involved conducting an integrative review, document analysis, and focus group development.

**Results:**

a specific situation theory was developed to describe and explain the transition experience of people with intestinal ostomies and their families, comprising seven concepts and 20 sub-concepts. The theoretical framework also includes relational statements, as well as a diagram representing its concepts and their interrelationships.

**Conclusion:**

the theory developed has the potential to guide and systematize nursing care for people with ostomies and their families, covering the different phases of the transition process.

## Highlights

**(1)** SST is a significant contribution to nursing knowledge. **(2)** It involves individual aspects and the family and social implications of the transition. **(3)** It has the potential to guide and systematize care throughout the transition. **(4)** It can support and guide the stages of the Nursing Process. **(5)** It can be applied to different healthcare settings.

## Introduction

Illness disrupts the relationship between human beings and their everyday world, being an unpredictable and significant event given its impact on personal, family, and community life^1^. It can be accompanied by varying degrees of physical dependence and multiple feelings, resulting in significant changes, postponing plans and projections for the future.

The unpredictability of events arising from illness alters the state of individual and family balance, necessitating reorganization in the face of the transition experienced^1^. Transition is the passage from one phase of life, condition, or *status* to another, resulting from complex interactions between people and the environment. It consists of moving from a stable state to another relatively stable state^2^.

Being sick represents a transition in people’s lives that is characterized as a unique, multidimensional, and complex experience, associated with periods of emotional instability, both in individual and family life, generating reactions and behaviors in the face of the experiences of illness^2^. This leads the person and/or their family to move through different phases, from a state of comfort, security, and tranquility to periods of imbalance, uncertainty, and conflict, which generate behavioral changes, aimed at finding stability^3^ ^-^ ^4^.

The diagnosis of a disease, accompanied by the creation of an intestinal ostomy, represents a significant transition process, generating ambivalent feelings that result in uncertainty, culminating in the need for new coping strategies to restore balance and well-being^5^. This reality entails changes in the family environment, as the care demands imposed by the disease and the ostomy’s presence affect other family members and shape family dynamics, thereby characterizing it as a collective event^6^ ^-^ ^8^.

The family is the primary social support network for people with ostomies and significantly influences rehabilitation through its participation in care activities^9^. Family support is a determining factor in the transition experience and can have positive or negative effects on the process of acceptance and adaptation to new living conditions^10^. The creation of an ostomy gives rise to new and specific care needs, promoting physical and physiological changes that also have repercussions for the psychosocial and spiritual dimensions^11^ ^-^ ^12^. 

Ostomy brings out the vulnerabilities and fragilities of the person and their family, which manifest themselves in organizational, emotional, and psychological implications, such as compromised abilities and capacities for daily tasks, distorted self-image due to bodily mutilation, fear, and insecurity that can lead to social isolation. Thus, nursing care must attend to changes that extend beyond physical and physiological dimensions, delving into the subjective aspects that accompany the transition process. Considering this, nurses can develop care strategies that contribute significantly to the construction of a positive experience^13^ ^-^ ^15^.

Given the complexity of nursing care for these individuals and their families, it is crucial to emphasize the need to deepen theoretical knowledge as an alternative to guide and qualify care. The development of theories that represent the transition experience can help characterize phenomena of interest to professionals caring for this population, thereby supporting the identification of human responses and the implementation of interventions grounded in the Nursing Process. Theoretical references that describe or explain this transition experience contribute to the paradigm of theory-guided practice and to the organization and systematization of scientific and philosophical knowledge in nursing^16^ ^-^ ^17^.

In this context, specific situation theories (SSTs) stand out for their ability to represent and describe a limited set of concepts, explaining their relationships and predicting outcomes based on these interrelationships. Thus, they are more specific, differing from other theories in their level of abstraction, scope, and context^18^.

The development of an SST enables a sensitive approach to nursing care, considers the multiple dimensions of the transition process experienced by people with ostomies and their families, and aims to organize comprehensive, unique, and integrated care, enabling the planning of specific actions consistent with the needs of this population. Considering the above, this study was based on the following research question: What constituent elements comprise a specific situation theory representative of the transition experience of people with intestinal ostomies and their families? The objective was to develop an SST that captures the transition experience of people with intestinal ostomies and their families, grounded in Afaf Meleis’ Transitions Theory (TT).

## Method

### Study design

Theoretical study, guided by the theory-research-theory strategy and based on an integrative approach for the development of a descriptive and explanatory SST^19^.

### Data collection

Deductive and inductive strategies were used to develop the SST. In the deductive stage, Afaf Meleis’ TT was used to provide the conceptual framework and organize theoretical thinking. Inductive strategies included developing an integrative literature review, document analysis, and qualitative research of a descriptive-exploratory nature.

### Deductive strategy

TT provided the basis for analogies that explained and facilitated understanding of the phenomenon of interest. Thus, the theoretical framework was developed through careful, thorough reading, which enabled the extraction of concepts and sub-concepts from TT that could contribute to representing the transition experience of the person with an intestinal ostomy and their family. The concepts deduced from the TT for the development of the SST were: types and patterns of transition; properties of the transition experience; transition conditions (facilitating and inhibiting); process indicators; outcome indicators; and nursing therapy. The concepts derived from the TT served as the basis for analyzing the scientific literature through an integrative review and documentary analysis. Thus, 28 codes corresponding to each concept and sub-concept derived from TT were created in Atlas.ti 22 software to facilitate data collection in later stages. The theoretical structure of TT also provided possible relational statements between the concepts that emerged during the theorization process.

### Inductive strategy: integrative literature review

The integrative review sought to broaden understanding of the transition experienced by people with ostomies and their families by analyzing scientific evidence. It was operationalized into six predefined stages: formulation of the review question; sampling of the literature; categorization of studies; evaluation of included studies; interpretation of results; and synthesis of knowledge.

Initially, the research topic and problem were defined, which allowed the following review question to be formulated: “What are the experiences of people with intestinal ostomies and their families in relation to illness and ostomy creation?”

Subsequently, searches were conducted in the following databases: Latin American and Caribbean Health Sciences Literature (LILACS); Brazilian Nursing Database (BDENF); Spanish Bibliographic Index in Health Sciences (IBECS); Medical Literature Analysis and Retrieval System Online (MEDLINE)/United States National Library of Medicine (PubMed); Cumulative Index to Nursing and Allied Health Literature (CINAHL); and Web of Science. Different search strategies were used in each database, combining different descriptors and keywords with Boolean operators, as detailed below:

First strategy: (estoma OR estomia OR ostoma OR ostomia OR “estomas cirúrgicos” OR colostomia OR ileostomia OR estomaterapia OR enterostomia) AND (família OR famílias OR familiares OR familiar OR “membros da família”) AND (experiência OR experiências OR vivência OR vivências OR impacto OR repercussão OR repercussões OR adaptação OR adaptações OR implicação OR implicações OR sentimentos OR emoções OR sexualidade OR aceitação OR reabilitação OR “qualidade de vida”).

Second strategy: ((ostomy OR colostomy OR ileostomy OR “surgical stomas”) AND (family OR families) AND (“life change events” OR experience OR experiences OR recovering OR adaptation OR implication OR feeling OR emotions OR sexuality OR acceptance OR “quality of life”)).

Studies in English, Portuguese, or Spanish were included, provided they were available in full and focused on the experiences of individuals and their families facing illness and intestinal ostomy. The exclusion criteria were: studies conducted with children and adolescents, or those addressing types of ostomies other than intestinal ostomies, as well as theses, dissertations, monographs, manuals, book chapters, experience reports, reflections, reviews, and editorials.

The searches yielded 1,490 studies, which were imported into EndNote Basic software. Of this total, 309 were excluded as duplicates, leaving 1,181 publications. The analysis of these references was carried out using Rayyan software, in which the titles and abstracts were initially reviewed, resulting in the exclusion of 1,094 studies that did not meet the established criteria. Thus, 87 studies were preselected and thoroughly reviewed. After careful reading, it was found that 48 studies did not address the review question, allowing the analysis to be limited to 39 articles. The bibliographic survey was conducted between March and June 2022 via the CAPES Journal Portal, in a double-blind independent study. When there were disagreements regarding the eligibility of publications, these were resolved through consensus and analysis by a third reviewer. It is important to note that the selection and analysis process for the articles followed the recommendations of the Preferred Reporting Items for Systematic Reviews and Meta-Analyses (PRISMA) protocol.

The data were interpreted considering TT, using inductive-deductive reasoning, with a view to identifying evidence related to the theory’s concepts, while considering the phenomenon under study. During the analysis, citations were created: segments of the text considered relevant and corresponding to the codes previously established in the deductive strategy, as well as free citations, which were later associated to create new codes.

This stage allowed us to apply inductive reasoning in the theorization process and to develop two new concepts and 11 sub-concepts based on the evidence identified in the literature. Thus, at the end of this stage, the structure of the emerging SST was expanded, yielding eight concepts and 33 sub-concepts, enabling the development of a theoretical structure capable of representing the experiences and lives of individuals and their families in relation to illness and ostomy creation.

### Inductive strategy: document analysis

In this stage, scientific publications on nursing care for people with intestinal ostomies and their families from the Center for Studies in Care and Family (NECFAM) at the Federal University of Santa Maria were analyzed. NECFAM publications on intestinal ostomies served as a data source for developing the SST, complementing the integrative theorization process.

Thirteen documents were analyzed, including research and extension reports, undergraduate monographs, master’s dissertations, and doctoral theses. The document analysis sought to answer the following review questions: “What are the life experiences and lessons of people with intestinal ostomies and their families in relation to illness and ostomy creation?” and “What nursing care actions are taken with people with intestinal ostomies and their families?”

Initially, this stage involved extracting data for summary charts, which were then exported to Atlas.ti 22 software. Subsequently, the evidence was analyzed and synthesized through interpretive reading considering TT, following inductive-deductive reasoning. From this, new citations were prepared, highlighting relevant segments of the text and corresponding to the codes for the concepts and sub-concepts of TT, as well as free citations, which were later associated with new codes.

The synthesis of the documentary analysis enabled the free codes to be linked to the groups of codes related to the concepts and sub-concepts of TT, as well as to the new concepts and sub-concepts identified in the previous stage. Thus, the theoretical structure developed up to the integrative review stage was not expanded, but the evidence identified in the document analysis provided greater theoretical density to the previously developed concepts, in addition to clarifying new aspects of the experiences and lives of individuals and their families related to illness and ostomy creation. At the end of this stage, it was possible to establish and confirm the relationships among SST concepts through abstraction, which considered scientific evidence and the theoretical structure of TT. Thus, a diagram representing the emerging theory was developed, signaling the concepts, sub-concepts, and their relationships, as well as the phases that comprise the transition process experienced by the person with an ostomy and their family.

### Inductive strategy: focus group

To strengthen, expand, refine, or refute the concepts and statements derived from the deduction of TT and induction, based on the findings of the literature review and document analysis, a qualitative, descriptive-exploratory study was developed using the focus group (FG) technique. The participants included six members of NECFAM due to their previous experience in research, teaching, and extension in the field of nursing care for people with intestinal ostomies and their families. The selection of participants was intentional, and the sample was considered adequate for the purpose of refining the theoretical framework developed, in which the depth of discussion and the expertise of the participants are more relevant than the search for saturation of new themes, which is consistent with the methodological guidelines for the operationalization of FG.

The selection criteria were: being a member of NECFAM, having at least six months of participation in projects on the topic, and having a scientific publication in the area. Members who were unfamiliar with the subject or did not regularly attend meetings were excluded.

Of the six participants, two were doctors, two were postgraduate students, one at the doctoral level and the other at the master’s level, and two were undergraduate research students. To operationalize the GF, a TSE presentation was prepared in digital format using Microsoft PowerPoint, and a summary of the theoretical framework (the presentation of the theory) was sent to participants in advance as a Microsoft Word document. In addition, a guide of topics was prepared to mediate the group discussions based on the concepts, sub-concepts, relational statements, and theory diagram, which also comprised the material organized for the presentation and summary sent previously. The guide addressed understanding the transition process, the nature and properties of concepts, the constraints of transition, as well as response patterns and their applicability in clinical practice. 

The GF meetings were organized in three stages: welcoming and integrating participants, collecting data from support material and group interaction, and summarizing and reaching consensus on what was discussed. Three GF meetings were held on different dates between July and October 2023, via Google Meet video conference. These meetings were recorded in audio and video, transcribed in full, and supplemented by a field diary.

At the first meeting, the general structure (concepts, sub-concepts, and their relationships) was discussed; at the second, the representative diagram was refined; and at the third, the final diagram was validated. Based on the FG meetings, it was possible to qualify and refine the theoretical structure and the representative diagram of the theory, thereby adding different perspectives to the phenomenon under study. Furthermore, the validity of participants’ contributions emerged through consensus and deliberations continually affirmed by the group, which served as the primary mechanism for ensuring the reliability of their contributions. This step followed the recommendations of the Consolidated Criteria for Reporting Qualitative Research (COREQ).

### Ethical aspects

In conducting this study, the ethical precepts of Resolution No. 510, dated April 7, 2016, of the National Health Council, which deals with guidelines and regulatory standards for research involving human subjects, were respected. Research approved by the Research Ethics Committee under opinion number 5,346,695.

## Results

The integration of the results from the deductive and inductive strategies culminated in the organization of seven concepts and 20 sub-concepts to describe and explain the transition experience of people with intestinal ostomies and their families, as shown in Figure 1.

**Figure 1:** Concepts and subconcepts that comprise the SST* of the transition experience of people with intestinal ostomies and their families. Santa Maria, RS, Brazil, 2024

Concept	Sub-concept
They notice changes in the body’s functionality	Identify and observe signs and symptoms;
	Encourage seeking help;
	Seek to expedite diagnosis.
They deny the illness and the ostomy	They delay seeking medical care;
	They delay undergoing surgery;
	They avoid looking at their body after the ostomy.
Experience types and patterns of transition	They confirm the diagnosis and face the unknown;
	They redefine roles and family organization considering the diagnosis and stoma.
Transition properties	They accept the new reality and collectively engage in caregiving;
	They acknowledge critical events and changes over time.
Particularities of the living environment as facilitators and/or inhibitors	Sociodemographic and clinical/surgical profile;
	Emotional significance attributed to the experience of illness and ostomy;
	Individual and family beliefs in the lived experience;
	Anticipate knowledge.
External factors as facilitators and/or inhibitors	Support received from health services and ostomy associations.
	Family in the context of transition;
	Affectionate interpersonal relationships;
	Aspects related to social interaction.
Answers that guide rehabilitation for living with a stoma	Cooperation in the pursuit of personal and family stability;
	Mastery of skills and behaviors for identity reformulation.

*SST = Specific Situation Theory

*They perceive changes in bodily functions* during the phase preceding the transition, when the first changes *are identified and observed through signs and symptoms*. This leads to mobilization within the family, *encouraging them to seek help* from health services and *expedite diagnosis*, resulting in a period marked by anxiety and fear. 

*They deny the illness and the ostomy* involves *delaying seeking care* out of fear of confirming the illness*.* Faced with an unwanted diagnosis, they reject the disease *to postpone surgery,* as there is insecurity and uncertainty about the ostomy procedure*.* This leads to dysfunctional behaviors in the postoperative period*,* such as *avoiding looking at the body after the ostomy,* which ultimately restricts the ability of the person and their family to adapt, as they are not yet aware of the transformation processes that signal the transition has not yet begun*.*

*They experience transition types and patterns* are related to *the confirmation of illness through diagnosis,* which leads to *confronting the unknown.* The therapeutic outcome is the creation of a stoma, which occurs unexpectedly and necessitates *redefining roles and simultaneously organizing intrafamilial resources.* Thus, it is understood that these people experience multiple and relational transitions that originate in a health-illness context, as a collective experience, with specific aspects for both the person and the family, but which are mutually influenced.

*Properties of transition* comprise awareness and perception of the transformations experienced, which result in *acceptance of the new reality*, *and thus cause the person and their family to become involved in care*, through the search for resources and strategies that strengthen the family unit. It encompasses behaviors related to transition, which are not static and defined, but rather the result of complex interactions between people and the environment. During this process, *changes and critical events occur* over time, generating repercussions and necessitating readjustments across various aspects, and the situation persists in an indefinite period of instability and vulnerability.

*Particularities of the life context as facilitators and/or inhibitors* involve *sociodemographic and clinical/surgical characteristics,* as well as the *ability to establish emotional meaning to the experience,* influenced by *individual and family beliefs* and the necessary preparation *to anticipate knowledge to experience the transition process*, which can limit or expand their therapeutic resources.

*External factors, such as facilitators and/or inhibitors,* relate to conditions and factors in the community and social environment that directly influence the lived experience*.* This refers *to the support received from health services and ostomy associations,* which constitute a resource that promotes favorable conditions for the new life situation*.* It also includes *the family’s involvement in the context of transition,* as well as *interpersonal relationships of affection,* which can either facilitate or inhibit the conditions of this process. *Aspects of social interaction* are directly affected by the burden of ostomy care demands, which may expose them to delicate situations that ultimately prevent them from meeting social expectations.

*Responses that guide rehabilitation to live with a stoma* involve a process experienced over time during the transition, with *cooperation and interaction being essential in the search for stability and personal and family well-being*. This requires *mastering new skills, competencies, and behaviors, with a view to developing a new identi*ty that allows for the continuity of life, grounded in a sense of normality and in the recognition of the changes resulting from the transition experienced. 

### Concepts and their relational statements

**Figure 2:**
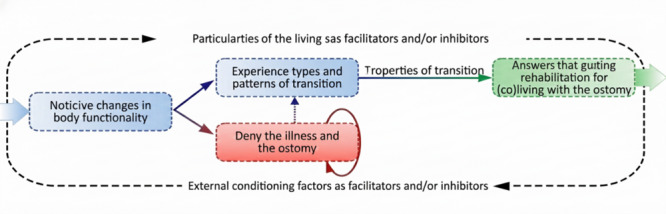
Diagram representing the concepts and relational statements of the TSE* of the transition experience of people with intestinal ostomies and their families. Santa Maria, RS, Brazil, 2024

The synthesis of the results enabled the selection of concepts and sub-concepts and the development of relational theoretical statements indicating relationships between two or more concepts, culminating in the creation of a diagram representing the theoretical structure and its statements, as shown in Figure 2.

*SST = Specific Situation Theory

*Perceiving changes in bodily* function is directly related to their experiences of types and patterns of transition, as it consciously precedes the onset of the lived experience and is influenced by the particularities of the life context, as well as by external factors that may facilitate or inhibit the transition. It is a condition that makes it possible to establish an opposition that denies illness and ostomy, since by recognizing changes in the body’s functional pattern, they can often deny what is being experienced.

*Denying the illness and ostomy* is influenced by the particularities of the life context and by external factors that affect the experience, that can make it possible or impossible enable the understanding of types and patterns of transition and, thus, keep the person with an ostomy and their family in a pre-transition phase, or else direct actions and responses that enable them to experience the transition, leaving the cycle of denial with some degree of awareness about the changes related to this process.

*Experiencing transition types and patterns* correlate with transition properties, which comprise essential characteristics that indicate awareness and acceptance of change, generate involvement, and confirm critical events throughout the process, while being influenced by the particularities of the life context and external constraints. This concept also modulates the responses that guide rehabilitation to live with the stoma, enabling intra-family reorganization and the redefinition of roles to restore stability and master the new reality of life.

The *properties of transition* exert influence, especially during the transition phase, which is not linear or static but rather the product of complex interactions among the person, their family, and their environment. It reflects awareness of what is being experienced, which affects their level of involvement and, in turn, inspires behaviors, reactions, and responses that guide rehabilitation. Furthermore, they are influenced by the particularities of the life context and external conditions, which can facilitate or restrict the process towards a healthy transition.

The *particularities of the life context, as facilitators and/or inhibitors,* involve individuals’ and families’ specificities, and are related to external constraints, that influence the conditions under which the transition occurs. Thus, these elements are present and resonate significantly throughout the transition experience, shaping how people move through the process. 

The *responses that guide rehabilitation to live with the ostomy* are related to experiencing types and patterns of transition, as they involve facing the unknown after diagnosis confirmation and the consequent redefinition of personal, family, and social roles. Furthermore, they are related to the properties of transition, being influenced by the particularities of the life context and external constraints.

## Discussion

In this study, the transition experience of people with ostomies and their families was anticipated by the first signs of illness, which were perceived and recognized. From this point on, there is intra-family mobilization, leading to seeking help from various public and/or private health services to speed up the diagnosis. Thus, the therapeutic journey of people with ostomies seeking specialized care is also evident, beginning with the discovery of physical changes, followed by access to health services to formalize the diagnosis and seek treatment^20^.

For families, the experience of living with an adult family member who has undergone ostomy due to intestinal cancer began with interaction with the sick family member, when they realized that something was wrong, as they observed signs of the disease in the form of functional changes in the intestine. Families understand that it is necessary to seek care from health services and encourage their family members to obtain answers about the signs and symptoms presented^8^.

In the transition experience, illness and ostomy arise unexpectedly and are perceived as a complex and traumatic event, which hinders adaptation and healthy transition. These changes redefine personal, social, and family roles and reorganize family resources. The family moves to cooperate, organize, and collaborate in care actions, especially at home, which favors acceptance and adaptation to the new living condition^21^. The configuration of a new family structure and dynamic is a way of facing and managing illness and the demands arising from the stoma^22^.

Accepting illness and ostomy as a therapeutic consequence can positively influence quality of life, alleviating daily challenges^23^. Furthermore, emotional responses are essential factors in acceptance and, consequently, in self-efficacy in care and self-care, helping overcome changes and influencing the ability to successfully rebuild the meaning of life^24^.

Initially, individuals and their families tend to deny the disease to avoid surgery, which results in dysfunctional behaviors in the postoperative period. A study conducted with 100 people with intestinal ostomies identified that the postoperative period is the most critical and painful stage, which compromises well-being and leads to an inability to control the unpleasant side effects of the ostomy, exacerbating feelings of rejection, resulting in social isolation and a delay in accepting the situation^25^. Living with an ostomy causes people to experience extreme emotions and negative feelings that dominate their lives before and after surgery, as it implies inevitable changes and is perceived as a critical moment. This new reality affects their physical and psychological condition, making them vulnerable and dependent, and altering their lifestyle and daily activities, with significant restrictions and changes in their personal and social lives^5^. Thus, the transitional period confirms the changes these people perceive, experience, and involves critical events that have repercussions on the biopsychosocial dimensions and directly modify their lifestyles.

Among the factors that influence the course of transition are the particularities of the life context as facilitators and/or inhibitors of the process, which encompasses personal conditions such as sociodemographic and clinical/surgical profile, emotional significance, beliefs, and preparation that anticipates knowledge and allows for the elaboration of the changes that will be experienced.

In a study^15^ conducted to identify the facilitators of the transition to self-care for people with ostomies, the following personal factors were identified: the ability to attribute positive meaning to the ostomy; the availability of guidance on care and life changes received during preoperative preparation; psychological stability; and comfort found in faith and religiosity.

It is known that the quality of life of people with ostomies is related to demographic factors such as age, marital status, place of residence, and education^26^. Advanced age increases difficulties with daily life with an ostomy and makes it harder to care for the skin and collection equipment. For single people, quality of life deteriorated rapidly after the ostomy. Place of residence (rural or urban) affects perceived quality of life, especially for those living in the countryside or in large cities, due to the difficulties of caring for the ostomy and the collection bag. The higher the level of education, the better the perceived quality of life after ostomy, and the longer the duration of ostomy, the more it directly interferes with this perception. Regarding clinical and surgical aspects, comorbidities, inadequate stoma location, and postoperative complications (early and late) are predictors that inhibit the transition process, as they hinder and compromise adaptation to the new living condition^27^.

These characteristics also influence the emotional significance attributed to the process of transformation and change, which are related to individual and family beliefs. Spirituality and faith are resources used to alleviate suffering, bringing comfort and hope^28^. By giving meaning to their experiences from a neutral or optimistic perspective, these people can reorganize themselves cooperatively to overcome difficulties and limitations, without giving significant importance to inconveniences, which directs their attitudes and behaviors toward well-being. When meanings and feelings are negative, they can inhibit adaptive mechanisms and response patterns, resulting in dysfunctional attitudes that generate instability and vulnerability within the family nucleus and compromise a healthy transition.

Given the above, implementing nursing interventions that increase positive response patterns, such as self-confidence, make them feel responsible and involved in the transition process and in continuing their lives, and help them access their personal resources (skills, self-efficacy) and/or external resources (informal and professional support networks)^3^. To this end, care must transcend the biological dimension, focusing on physical and physiological changes and paying attention to biopsychosocial aspects, through a care plan tailored to their needs that values the changes arising from their new living conditions^29^.

The support received from family, health services, ostomy associations, interpersonal relationships, as well as aspects related to social interaction, constitutes external resources available in the community and social environment and influences the experience, as they facilitate or inhibit the transition process^24^. When family and marital relationships are fragile, they can negatively impact the acceptance and adaptation of the person with an ostomy^9^ ^,^ ^30^.

Community health services support have a positive impact on the lives of people with ostomies and their families. Support from the state and the health system enables continuity of care and education after hospital discharge, with an emphasis on nursing interventions that aim to alleviate the adverse effects of the ostomy, reduce stress, and address problems to improve quality of life^5^.

Nursing interventions promote personal and family potential, which is essential for self-care and independence. The transition to self-care is a central element in the experience of people with ostomies in terms of their rehabilitation. It is the result of a complex transformation process that involves mastering new skills and competencies to mitigate critical events and facilitate behavioral change. By reducing the adaptation process to the specificities of these people’s experience, it is possible to shift perspectives towards healthier transitions, leading to responses that guide rehabilitation towards living with the ostomy.

However, it is imperative to recognize the theory’s limitations, given its restricted scope and level of abstraction. As the theory is integrated into clinical practice, its ongoing validation and refinement are essential to ensure its long-term effectiveness and relevance. The suggestion of the need for external validation does not limit its applicability, as it is necessary to test and apply the theory across different care contexts for people with ostomies and their families. This validation will not just strengthen its theoretical framework; it will contribute to its ongoing development.

Future studies are recommended to apply and validate this theory in clinical practice, testing it across different care settings and directly with people with intestinal ostomies and their families, to assess its effectiveness in guiding nursing interventions and promoting healthy transitions. External validation, both by specialist nurses and the target population, will not only strengthen its theoretical framework, but also contribute to its continued development and refinement.

## Conclusion

It was possible to develop an SST representative of the transition experience of people with intestinal ostomies and their families. Given the integrative approach adopted in this study, the development of the SST is a significant contribution to nursing knowledge, particularly regarding the care of people with intestinal ostomies and their families. Its theoretical structure presents seven concepts that describe and explain the transition experience of these individuals, providing a comprehensive, holistic view of a complex, multifaceted process that examines not only individual aspects, but also family and social implications.

Furthermore, it shows that adapting to an intestinal ostomy is not limited to physical and physiological changes in the body, but also involves reconfiguring the person and their identity, relationships, and responsibilities within and outside the family. The redefinition of roles within the family unit, driven by this transition, highlights the importance of intervention strategies that consider not only the person with the ostomy, but also family members as active participants in the adaptation process.

It is essential to emphasize that theory provides a consistent theoretical basis that supports and guides the stages of PE through standardized language, assisting clinical reasoning and decision-making. Thus, it has the potential to significantly improve nursing care, as it is grounded in scientific and empirical evidence, enabling the organization of a *continuum* of comprehensive preventive and therapeutic actions that are sensitive to the needs of this population, thereby promoting a practical, thorough approach. 

The theory not only informs practice, but also guides the creation of operational protocols, flowcharts, and other methodological instruments that can assist nursing care. These tools can contribute to the systematization of care, ensuring a consistent and qualified approach across different healthcare settings, highlighting their practical applicability.

## Data Availability

Datasets related to this article will be available upon request to the corresponding author.
