# Supplementary material for: Specific situation theory of the transition experience lived by people with intestinal ostomies and their families
Source: Rev Lat Am Enfermagem. 2026 Jun 15;34:e4858. doi: 10.1590/1518-8345.7979.4858 (PMC13277349; doi:10.1590/1518-8345.7979.4858)
Supplement: Translation

# Translation: Teoria de situação específica da experiência de transição vivida pela pessoa com estomia intestinal e sua família*

**DOI:** 10.1590/1518-8345.7979.4859

**Authors:** Angélica Dalmolin, Eduardo da Silva Gomes, Juliano Teixeira Moraes, Leonardo Rozinelli, Maria Ribeiro Lacerda, Nara Marilene Oliveira Girardon-Perlini

**Affiliations:** 1 Universidade Federal de Santa Maria, Enfermagem, Santa Maria, RS, Brazil.; 2 Scholarship holder at the Coordenação de Aperfeiçoamento de Pessoal de Nível Superior (CAPES), Brazil.; 3 Scholarship holder at the Conselho Nacional de Desenvolvimento Científico e Tecnológico (CNPq), Brazil.; 4 Universidade Federal de São João del-Rei, Enfermagem, Minas Gerais, MG, Brazil.; 5 Universidade Federal de Santa Maria, Medicina, Santa Maria, RS, Brazil.; 6 Universidade Federal do Paraná, Enfermagem, Curitiba, PR, Brazil.

**Keywords:** Estomia, Família, Cuidados de Enfermagem, Teoria de Enfermagem, Estomaterapia, Modelos de Enfermagem

## Abstract

**Objetivo:**

desenvolver uma teoria de situação específica representativa da experiência de transição vivida pelas pessoas com estomia intestinal e suas famílias, a partir da Teoria das Transições de Afaf Meleis.

**Método:**

estudo teórico, guiado pela estratégia teoria-pesquisa-teoria e fundamentado na abordagem integrativa, a qual conjuga estratégias dedutivas e indutivas. Na etapa dedutiva, elencou-se a Teoria das Transições para fornecer a estrutura conceitual e organizar o pensamento teórico. As estratégias indutivas envolveram a realização de uma revisão integrativa, análise documental e o desenvolvimento de grupo focal.

**Resultados:**

desenvolveu-se uma teoria de situação específica que descreve e explica a experiência de transição vivida pelas pessoas com estomia intestinal e sua família por meio de sete conceitos e 20 subconceitos. A estrutura teórica também engloba as afirmações relacionais, além de um diagrama representativo dos seus conceitos e suas interrelações.

**Conclusão:**

a teoria desenvolvida tem potencial para guiar e sistematizar o cuidado de enfermagem à pessoa com estomia e sua família, considerando as diferentes fases do processo de transição.

## Destaques

**(1)** A TSE é uma contribuição significativa para o conhecimento da enfermagem. **(2)** Envolve aspectos individuais e as implicações familiares e sociais da transição. **(3)** Tem potencial para guiar e sistematizar o cuidado ao longo da transição. **(4)** É capaz de sustentar e orientar as etapas do Processo de Enfermagem. **(5)** Pode ser aplicada aos diferentes cenários de atenção à saúde.

## Introdução

O adoecimento suscita rupturas na relação do ser humano com o seu mundo cotidiano, sendo um evento imprevisível e significativo ante o impacto na vida pessoal, familiar e comunitária^1^. Pode ser acompanhado de diferentes graus de dependência física e múltiplos sentimentos, resultando em alterações significativas, postergando os planos e as projeções para o futuro.

A imprevisibilidade dos fatos decorrentes do adoecimento altera o estado de equilíbrio individual e familiar, resultando na necessidade de reorganização face à transição experienciada^1^. A transição é a passagem de uma fase da vida, condição ou *status* para outra, sendo resultado de interações complexas entre pessoas e o ambiente. Consiste em passar de um estado estável para outro estado relativamente estável^2^. 

Estar doente representa na vida das pessoas uma transição que se caracteriza como uma vivência única, multidimensional e complexa, associada a períodos de instabilidade emocional, tanto na vida individual quanto na vida familiar, gerando reações e comportamentos em face da experiência do adoecimento^2^. Isso conduz a pessoa e/ou sua família a mover-se por diferentes fases, passando de uma condição de conforto, segurança e tranquilidade a períodos de desequilíbrio, incertezas e conflitos, o que gera mudanças de comportamento, a fim de encontrar a estabilidade^3^ ^-^ ^4^.

O diagnóstico de uma doença, acompanhado da confecção de uma estomia intestinal, representa um processo de transição marcante, gerando uma ambivalência de sentimentos que resulta em incertezas, culminando na necessidade de novas estratégias de enfrentamento, a fim de restabelecer o equilíbrio e bem-estar^5^. Essa realidade implica em mudanças no âmbito familiar, pois as situações de cuidado impostas pela doença e a presença da estomia refletem sobre os demais membros da família e impactam a dinâmica familiar, caracterizando-se como um evento coletivo^6^ ^-^ ^8^.

A família é o principal núcleo de relação interpessoal da pessoa com estomia e exerce influência significativa na reabilitação por meio da sua coparticipação nas ações de cuidado^9^. O apoio familiar é fator determinante à experiência de transição, podendo refletir de forma positiva ou negativa no processo de aceitação e adaptação à nova condição de vida^10^. A confecção da estomia faz emergir necessidades novas e específicas de cuidado, ao promover alterações físicas e fisiológicas, repercutindo, ainda, nas dimensões psicossociais e espirituais^11^ ^-^ ^12^. 

A estomia faz emergir as vulnerabilidades e fragilidades da pessoa e da família, as quais se manifestam em implicações de desordem organizacional, emocional e psicológica, como o comprometimento de habilidades e capacidades para as tarefas diárias, a distorção da autoimagem pela mutilação do corpo, o medo e a insegurança que podem levar ao isolamento social. Deste modo, é necessário que o cuidado de enfermagem envolva atenção em meio às alterações que transpõem as dimensões físicas e fisiológicas, adentrando os aspectos subjetivos que acompanham o processo de transição vivenciado. Em vista disso, o enfermeiro pode tecer estratégias de cuidado, a fim de contribuir de maneira significativa na construção de uma vivência e experiência positiva^13^ ^-^ ^15^.

Ao considerar a complexidade que envolve o cuidado de enfermagem a estas pessoas e suas famílias, é válido ressaltar a necessidade de aprofundar conhecimentos teóricos como alternativa para orientar e qualificar o cuidado. O desenvolvimento de teorias representativas da experiência de transição pode colaborar para a caracterização dos fenômenos de interesse dos profissionais no cuidado a essa população, sustentando a identificação de respostas humanas e a implementação de intervenções com base no Processo de Enfermagem. Referenciais teóricos que descrevem ou explicam essa experiência de transição contribuem com o paradigma da prática guiada por teorias e com a organização e sistematização do conhecimento científico e filosófico da enfermagem^16^ ^-^ ^17^.

Nesse contexto, as teorias de situação específica (TSE) destacam-se por possuírem a capacidade de representar e descrever um conjunto limitado de conceitos, explicando a relação entre eles, a fim de elaborar uma previsão de resultados a partir dessa inter-relação. Dessa forma, são mais específicas, sendo diferenciadas das demais teorias pelo seu nível de abstração, escopo e contexto^18^.

A elaboração de uma TSE possibilita um olhar sensível em relação ao cuidado de enfermagem, considerando as múltiplas dimensões que abarcam o processo de transição experienciado pela pessoa com estomia e sua família, visando à organização de um cuidado abrangente, singular e integral, possibilitando planejar ações específicas e congruentes às necessidades dessa população. Diante do exposto, este estudo embasou-se na seguinte questão de pesquisa: quais elementos constitutivos integram uma teoria de situação específica representativa da experiência de transição vivida pelas pessoas com estomia intestinal e suas famílias? Com isto, objetivou-se desenvolver uma TSE representativa da experiência de transição vivida pelas pessoas com estomia intestinal e a sua família, considerando a Teoria das Transições (TT) de Afaf Meleis. 

## Método

### Delineamento do estudo

Estudo teórico, guiado pela estratégia teoria-pesquisa-teoria e fundamentado na abordagem integrativa para o desenvolvimento de uma TSE, do tipo descritiva e explicativa^19^.

### Coleta de dados

Empregaram-se estratégias dedutivas e indutivas para o desenvolvimento da TSE. Na etapa dedutiva, elencou-se a TT de Afaf Meleis para fornecer a estrutura conceitual e organizar o pensamento teórico. As estratégias indutivas compreenderam o desenvolvimento de uma revisão integrativa da literatura, análise documental e uma pesquisa qualitativa, de natureza descritiva-exploratória.

### Estratégia dedutiva

A TT forneceu a base da analogia para as explicações e entendimentos sobre o fenômeno de interesse. Sendo assim, realizou-se a aproximação com o referencial teórico por meio da leitura minuciosa e criteriosa, o que tornou possível extrair conceitos e subconceitos da TT que pudessem contribuir para a representação da experiência de transição vivida pela pessoa com estomia intestinal e sua família. Os conceitos deduzidos da TT para o desenvolvimento da TSE foram: tipos e padrões de transição; propriedades de experiência de transição; condições de transição (facilitadoras e inibidoras); indicadores de processo; indicadores de resultado; e terapêutica de enfermagem. Os conceitos derivados da TT constituíram-se na base para a análise da literatura científica por meio de revisão integrativa e análise documental. Dessa forma, foram criados 28 códigos correspondentes a cada conceito e subconceito derivado da TT no *software* Atlas.ti 22, a fim de facilitar a coleta dos dados nas etapas posteriores. A estrutura teórica da TT também forneceu possíveis afirmativas relacionais entre os conceitos que emergiram durante o processo de teorização. 

### Estratégia indutiva: revisão integrativa da literatura

A revisão integrativa buscou ampliar a compreensão sobre a transição experienciada pela pessoa com estomia e sua família por meio da análise de evidências científicas. Foi operacionalizada considerando seis etapas pré-definidas, a saber: formulação da questão de revisão; amostragem da literatura; categorização dos estudos; avaliação dos estudos incluídos; interpretação dos resultados e síntese do conhecimento. 

Inicialmente definiu-se o tema e o problema de pesquisa, o que permitiu elaborar a seguinte pergunta de revisão: “Quais as experiências e vivências das pessoas com estomia intestinal e de sua família em relação ao adoecimento e à confecção do estoma?”. 

Posteriormente, foram realizadas buscas nas bases de dados: Literatura Latino-Americana e do Caribe em Ciências da Saúde (LILACS); Base de Dados de Enfermagem Brasileira (BDENF); Índice Bibliográfico *Español en Ciencias de la Salud* (IBECS); *Medical Literature Analysis and Retrieval System Online* (MEDLINE)/*United States National Library of Medicine* (PubMed); *Cumulative Index to Nursing and Allied Health Literature* (CINAHL); e *Web of Science*. Foram empregadas diferentes estratégias de busca em cada base de dados, combinando diferentes descritores e palavras-chaves com operadores booleanos, conforme detalhado a seguir: 

Primeira estratégia: (estoma OR estomia OR ostoma OR ostomia OR “estomas cirúrgicos” OR colostomia OR ileostomia OR estomaterapia OR enterostomia) AND (família OR famílias OR familiares OR familiar OR “membros da família”) AND (experiência OR experiências OR vivência OR vivências OR impacto OR repercussão OR repercussões OR adaptação OR adaptações OR implicação OR implicações OR sentimentos OR emoções OR sexualidade OR aceitação OR reabilitação OR “qualidade de vida”). 

Segunda estratégia: ((ostomy OR colostomy OR ileostomy OR “surgical stomas”) AND (family OR families) AND (“life change events” OR experience OR experiences OR recovering OR adaptation OR implication OR feeling OR emotions OR sexuality OR acceptance OR “quality of life”)).

Foram incluídos estudos em inglês, português ou espanhol, disponíveis na íntegra e que tivessem como objeto de pesquisa as experiências e as vivências das pessoas e de suas famílias ante o adoecimento e a confecção da estomia intestinal. Os critérios de exclusão foram: estudos realizados com crianças e adolescentes, ou ainda, aqueles que abordam outros tipos de estomia, que não as intestinais, além de teses, dissertações, monografias, manuais, capítulos de livros, relatos de experiência, reflexões, revisões e editoriais.

A partir das buscas, obtiveram-se 1.490 estudos, os quais foram importados para o *software* EndNote Basic. Desse total, foram excluídos 309 por estarem duplicados, resultando em 1.181 publicações. A análise dessas referências foi operacionalizada no *software* Rayyan, em que inicialmente foi realizada a leitura dos títulos e resumos, resultando na exclusão de 1.094 pesquisas por não atenderem aos critérios estabelecidos. Assim, 87 estudos foram pré-selecionados e lidos na íntegra. A partir da leitura minuciosa, verificou-se que 48 pesquisas não respondiam à questão de revisão, o que possibilitou delimitar o *corpus* de análise em 39 artigos. O levantamento bibliográfico foi realizado entre os meses de março e junho de 2022 via Portal de Periódicos da CAPES, de modo duplo independente. Quando houve divergências quanto à elegibilidade das publicações, estas foram resolvidas por meio de consenso e análise de um terceiro revisor. É importante destacar que o processo de seleção e análise dos artigos seguiu as recomendações do protocolo *Preferred Reporting Items for Systematic Reviews and Meta-Analyses* (PRISMA). 

A interpretação dos dados foi realizada à luz da TT, seguindo o raciocínio indutivo-dedutivo, com vistas a identificar as evidências relacionadas aos conceitos da teoria, considerando o fenômeno em estudo. Com a análise, foram criadas citações, ou seja, segmentos do texto considerados relevantes e que correspondessem aos códigos previamente estabelecidos durante a estratégia dedutiva, bem como elaboraram-se citações livres, as quais posteriormente foram associadas para criação de novos códigos. 

Essa etapa permitiu conjugar o raciocínio indutivo no processo de teorização e conformar dois novos conceitos e 11 subconceitos, embasados nas evidências identificadas na literatura. Assim, ao final dessa etapa, ampliou-se a estrutura da TSE emergente, conformando oito conceitos e 33 subconceitos, o que foi possível organizar uma estrutura teórica capaz de representar as experiências e vivências das pessoas e de sua família em relação ao adoecimento e à confecção da estomia.

### Estratégia indutiva: análise documental

Nessa etapa, foram analisadas as produções científicas relativas à temática do cuidado de enfermagem à pessoa com estomia intestinal e sua família do Núcleo de Estudos em Cuidado e Família (NECFAM) da Universidade Federal de Santa Maria. As produções do NECFAM relativas à temática das estomias intestinais consistiram em fonte de dados para o desenvolvimento da TSE, complementando o processo integrativo de teorização. 

Foram analisados 13 documentos, provenientes de relatórios de pesquisa e extensão; monografias de conclusão de graduação; dissertações de mestrado e tese de doutorado. A análise documental buscou responder às seguintes perguntas de revisão: “Quais as experiências e vivências das pessoas com estomia intestinal e de sua família em relação ao adoecimento e à confecção da estomia?”, e “Quais as ações de cuidado de enfermagem junto às pessoas com estomia intestinal e sua família?”. 

Inicialmente essa etapa foi operacionalizada por meio da extração dos dados para quadros sinópticos, os quais foram exportados para o *software* Atlas.ti 22. Posteriormente, realizou-se a análise e síntese das evidências por meio da leitura interpretativa à luz da TT, seguindo o raciocínio indutivo-dedutivo. A partir disso, foram elaboradas novas citações, sendo destacados nos documentos segmentos do texto considerados relevantes e que correspondessem aos códigos referentes aos conceitos e subconceitos da TT, bem como citações livres, as quais posteriormente foram associadas a novos códigos.

A síntese da análise documental possibilitou que os códigos livres fossem vinculados aos grupos de códigos relacionados aos conceitos e subconceitos da TT, bem como aos novos conceitos e subconceitos identificados na etapa anterior. Dessa forma, a estrutura teórica desenvolvida até a etapa de revisão integrativa não foi ampliada, mas as evidências identificadas na análise documental proporcionaram maior densidade teórica aos conceitos previamente elaborados, além de esclarecer aspectos novos relacionados às experiências e vivências das pessoas e de sua família em relação ao adoecimento e à confecção da estomia. Ao final dessa etapa, foi possível estabelecer e confirmar as relações entre os conceitos da TSE por meio de um processo de abstração, o qual considerou as evidências científicas e a estrutura teórica da TT. Assim, elaborou-se um diagrama representativo da teoria emergente, sinalizando os conceitos, subconceitos e suas relações e, ainda, as fases que compreendem o processo de transição experienciado pela pessoa com estomia e sua família.

### Estratégia indutiva: grupo focal

Com o objetivo de fortalecer, ampliar, refinar ou refutar os conceitos e as afirmações elaboradas a partir da dedução da TT e da indução, por meio dos achados da revisão de literatura e da análise documental, foi desenvolvido um estudo qualitativo, de natureza descritiva-exploratória**,** utilizando a técnica de grupo focal (GF). Os participantes compreenderam seis integrantes do NECFAM por suas experiências prévias em pesquisas, ensino e extensão na temática do cuidado de enfermagem às pessoas com estomia intestinal e sua família. A seleção dos participantes foi realizada de modo intencional e considerou-se a amostra adequada para o propósito de refinar a estrutura teórica desenvolvida, em que a profundidade da discussão e a expertise dos participantes são mais relevantes do que a busca por saturação de novos temas, o que coaduna com as orientações metodológicas de operacionalização de GF.

Os critérios de seleção foram: ser integrante do NECFAM, ter no mínimo seis meses de participação em projetos sobre a temática e possuir publicação científica na área. Foram excluídos membros sem aproximação com o tema ou que não frequentavam as reuniões assiduamente. 

Dos seis participantes, dois eram doutores, dois eram pós-graduandos, um a nível de doutorado e o outro de mestrado, e dois eram estudantes de iniciação científica. Para operacionalizar o GF, foi elaborada uma apresentação da TSE em recurso digital no Microsoft PowerPoint e encaminhada com antecedência aos participantes a síntese da estrutura teórica (apresentação da teoria) em um documento de texto no Microsoft Word. Ainda, elaborou-se um guia de temas para mediar as discussões do grupo com base nos conceitos, subconceitos, nas afirmações relacionais e no diagrama da teoria, os quais compunham também o material organizado para a apresentação e a síntese enviada previamente. O guia abordava a compreensão do processo de transição, a natureza e as propriedades dos conceitos, os condicionantes da transição, bem como os padrões de resposta e sua aplicabilidade na prática clínica. 

Os encontros de GF foram operacionalizados em três momentos: acolhimento e integração entre os participantes, coleta de dados a partir do material de apoio e da interação grupal, síntese e consenso do que foi deliberado. Foram desenvolvidos três encontros de GF em datas distintas, os quais ocorreram entre julho e outubro de 2023, sendo realizados por meio de videoconferência, com o auxílio da plataforma digital Google *Meet*, os quais foram gravados em áudio e vídeo, transcritos na íntegra e complementados por um diário de campo.

No primeiro encontro, discutiu-se a estrutura geral (conceitos, subconceitos e suas relações); no segundo, refinou-se o diagrama representativo; e no terceiro validou-se o diagrama final. A partir dos encontros do GF, foi possível qualificar e refinar a estrutura teórica e o diagrama representativo da teoria, o que acrescentou visões diferentes ao fenômeno estudado. Além disso, a validade das contribuições dos participantes emergiu por meio do consenso e das deliberações continuamente afirmadas pelo grupo, o que serviu como principal mecanismo para garantir a fidedignidade das contribuições. Esta etapa seguiu as recomendações do *Consolidated Criteria for Reporting Qualitative Research* (COREQ).

### Aspectos éticos

Para desenvolver este estudo, foram respeitados os preceitos éticos da Resolução n.º 510, de 07 de abril de 2016, do Conselho Nacional de Saúde, que trata das diretrizes e normas regulamentadoras de pesquisa com seres humanos. Pesquisa aprovada por Comitê de Ética em Pesquisa sob parecer número 5.346.695.

## Resultados

A integração dos resultados oriundos das estratégias dedutivas e indutivas culminou na organização de sete conceitos e 20 subconceitos para descrever e explicar a experiência de transição das pessoas com estomia intestinal e sua família, conforme visualizado na Figura 1.

**Figura 1:** Conceitos e subconceitos que compõem a TSE* da experiência de transição das pessoas com estomia intestinal e sua família. Santa Maria, RS, Brasil, 2024

Conceito	Subconceito
Percebem as mudanças na funcionalidade do corpo	Identificam e observam os sinais e sintomas;
	Incentivam a buscar ajuda;
	Buscam agilizar o diagnóstico.
Negam o adoecimento e a estomia	Postergam a procura por atendimento;
	Protelam a realização da cirurgia;
	Evitam olhar o corpo após a estomia.
Experienciam tipos e padrões de transição	Confirmam o diagnóstico e enfrentam o desconhecido;
	Redefinem os papéis e a organização familiar diante do diagnóstico e da estomia.
Propriedades da transição	Aceitam a nova realidade e envolvem-se coletivamente para o cuidado;
	Confirmam os acontecimentos críticos e as mudanças ao longo do tempo.
Particularidades do contexto de vida como facilitadores e/ou inibidores	Perfil sociodemográfico e clínico/cirúrgico;
	Significado emocional atribuído ao vivido com o adoecimento e a estomia;
	Crenças individuais e familiares na experiência vivida;
	Antecipam conhecimentos.
Condicionantes externos como facilitadores e/ou inibidores	Apoio recebido nos serviços de saúde e na associação de pessoas com estomia;
	Família no contexto de transição;
	Relações interpessoais de afeto;
	Aspectos relativos à convivência social.
Respostas que direcionam a reabilitação para (con)viver com a estomia	Cooperação na busca por estabilidade pessoal e familiar;
	Domínio de habilidades e comportamentos para reformulação da identidade.

*TSE = Teoria de Situação Específica

*Percebem as mudanças na funcionalidade do corpo* compreende a fase que antecede o processo de transição, momento em que as primeiras mudanças na funcionalidade do corpo são *identificadas e observadas* por meio dos *sinais e sintomas.* Assim, ocorre uma mobilização intrafamiliar que resulta em *incentivo para procurar ajuda* nos serviços de saúde e *buscar agilizar o diagnóstico*, o que resulta em um período permeado por ansiedade e medo. 

*Negam o adoecimento e a estomia* envolve o *adiamento na procura por atendimento*, em razão do receio de confirmar o adoecimento. Ante o diagnóstico indesejado, rejeitam a doença na tentativa de *protelar a cirurgia*, pois há insegurança e incerteza quanto à confecção da estomia. Isso implica comportamentos disfuncionais no pós-operatório, como *evitar olhar o corpo após a estomia*, o que acaba por restringir a capacidade de adaptação da pessoa e da sua família, pois ainda não estão conscientes dos processos de transformação, o que sinaliza que a transição ainda não começou. 

*Experienciam tipos e padrões de transição* relacionam-se com a *confirmação do adoecimento pelo diagnóstico,* o que resulta no *enfrentamento do desconhecido*. O desfecho terapêutico é a confecção da estomia, a qual se apresenta de forma inesperada e gera necessidade de *redefinir os pap*é*is e organizar, simultaneamente, os recursos intrafamiliares.* Desse modo, compreende-se que essas pessoas vivenciam transições múltiplas e relacionais que se originam por uma natureza de saúde-doença, sendo uma experiência coletiva, com aspectos específicos tanto para a pessoa quanto para a família, mas que são influenciados mutuamente. 

*Propriedades da transição* compreendem a consciência e percepção das transformações vividas, o que resulta na *aceitação da nova realidade*, *e assim, faz com que a pessoa e sua família venham a envolver-se no cuidado*, por meio da busca por recursos e estratégias que propiciem fortalecer o núcleo familiar. Engloba os comportamentos relacionados à transição, os quais não são estáticos e definidos, mas consequência de interações complexas entre as pessoas e o ambiente. Durante esse processo, *mudanças e acontecimentos críticos são vivenciados* ao longo do tempo, o que gera repercussões e implica reajustes em diversos aspectos e perpassa por um período indefinido de instabilidade e vulnerabilidade. 

*Particularidades do contexto de vida como facilitadores e/ou inibidores* envolve as *características sociodemográficas e clínico/cirúrgicas,* bem como a *capacidade de estabelecer significado emocional ao vivido*, tendo influência das *crenças individuais e familiares* e da preparação necessária ao *antecipar conhecimentos para experienciar o processo de transição*, o que pode limitar ou ampliar os seus recursos terapêuticos. 

*Condicionantes externos como facilitadores e/ou inibidores* relacionam-se com as condições e os fatores do ambiente comunitário e social que interferem diretamente na experiência vivida. Refere-se ao *apoio recebido nos serviços de saúde e na associação de pessoas com estomia*, o qual constitui-se como um recurso que promove condições favoráveis à nova condição de vida. Ainda, compreende o *envolvimento da família no contexto de transição*, bem como *as relações interpessoais de afeto*, as quais podem tanto facilitar quanto inibir as condições desse processo. Os *aspectos relativos à convivência social* são diretamente afetados em detrimento das demandas de cuidado com a estomia, o que pode vir a expô-los a situações delicadas que acabam não permitindo suprir as expectativas sociais. 

*Respostas que direcionam a reabilitação para (con)viver com a estomia* envolve o processo experienciado ao longo do tempo de transição, sendo a *cooperação e a interação essenciais na busca pela estabilidade e bem-estar pessoal e familiar.* Isso requer alcançar o *domínio de novas habilidades, competências e comportamentos,* com vistas *a reestruturar uma nova identidade* que permita a continuidade da vida, embasada no senso de normalidade e que reconheça as mudanças resultantes da transição vivida. 

### Conceitos e suas afirmações relacionais

A síntese dos resultados possibilitou estabelecer a seleção dos conceitos e subconceitos e elaborar afirmações teóricas relacionais, a partir da indicação das relações entre dois ou mais conceitos, o que culminou na criação de um diagrama representativo da estrutura teórica e suas afirmações, conforme a Figura 2. 

*Percebem as mudanças na funcionalidade do corpo* está diretamente relacionado com experienciam tipos e padrões de transição, pois antecede conscientemente o início da experiência vivida e é influenciado pelas particularidades do contexto de vida, bem como pelos condicionantes externos que podem facilitar ou inibir a transição. É uma condição que possibilita estabelecer uma oposição, negam o adoecimento e a estomia, visto que ao reconhecer as mudanças no padrão de funcionalidade do corpo, muitas vezes, podem negar o que está sendo vivido. 

*Negam o adoecimento e a estomia* é influenciado pelas particularidades do contexto de vida e pelos condicionantes externos que influenciam a experiência, o que pode (im)possibilitar experienciar tipos e padrões de transição e, assim, manter a pessoa com estomia e sua família em uma fase pré-transição, ou então, direcionar ações e respostas que possibilitem experienciar a transição, saindo do ciclo de negação com algum grau de consciência sobre as mudanças relativas a esse processo. 

**Figura 2:** Diagrama representativo dos conceitos e afirmações relacionais da TSE* da experiência de transição das pessoas com estomia intestinal e sua família. Santa Maria, RS, Brasil, 2024

*TSE = Teoria de Situação Específica

*Experienciam tipos e padrões de transição* correlacionam-se com as propriedades da transição, as quais compreendem características essenciais que indicam a consciencialização e a aceitação das mudanças, o que gera envolvimento e confirma acontecimentos críticos ao longo do processo, tendo interferência das particularidades do contexto de vida e dos condicionantes externos. Esse conceito também modula as respostas que direcionam a reabilitação para (con)viver com a estomia, haja vista que possibilita a reorganização intrafamiliar e a redefinição de papéis, a fim de restabelecer a estabilidade e dominar a nova realidade de vida. 

As *propriedades da transição* exercem influência, especialmente, na fase de passagem, a qual não é linear e estática, mas produto de interações complexas da pessoa consigo mesma, com sua família e com o ambiente. É reflexo da consciência acerca do que está sendo vivido, o que repercute no seu nível de envolvimento e, assim, inspira comportamentos, reações e respostas que direcionam a reabilitação. Ainda, sofrem interferência das particularidades do contexto de vida e dos condicionantes externos, os quais são recursos que podem facilitar ou restringir o processo em direção a uma transição saudável. 

As *particularidades do contexto de vida como facilitadores e/ou inibidores* envolvem as especificidades individuais e familiares relacionadas à experiência e estão relacionadas com os *condicionantes externos*, o que influencia as condições sob as quais ocorrem a transição. Assim, são elementos que se fazem presentes e ressoam significativamente em toda a experiência de transição, pois influenciam como essas pessoas se movem ao longo do processo. 

As *respostas que direcionam a reabilitação para (con)viver com a estomia* estão relacionadas com experienciam tipos e padrões de transição, pois implicam o enfrentamento do desconhecido com a confirmação do diagnóstico e a consequente redefinição dos papéis pessoais, familiares e sociais. Ainda, relacionam-se com as propriedades da transição, sendo influenciadas pelas particularidades do contexto de vida e pelos condicionantes externos.

## Discussão

Neste estudo, a experiência de transição das pessoas com estomia e sua família foi antecipada pelos primeiros indícios do adoecimento, o qual se deu pela percepção e reconhecimento dos primeiros sinais e sintomas. A partir disso, tem-se uma mobilização intrafamiliar que resulta na procura por ajuda nos diferentes serviços de saúde, públicos e/ou privados, como uma forma de agilizar o diagnóstico. Assim, também se evidencia o itinerário terapêutico de pessoas com estomia na procura por cuidados especializados, iniciado com a descoberta das alterações de ordem física, seguido do acesso aos serviços de saúde como forma de formalizar o diagnóstico e buscar o tratamento^20^.

Para as famílias, a origem da experiência de conviver com um familiar adulto com estomia por câncer intestinal deu-se a partir da interação com o membro da família adoecido, quando percebem que algo não está bem, pois observam sinais da doença na manifestação de alterações funcionais no intestino. As famílias entendem que é necessário buscar atendimento nos serviços de saúde e incentivam seu familiar a obter respostas acerca dos sinais e sintomas apresentados^8^.

Na experiência de transição, a doença e a estomia surgem de forma inesperada, sendo percebidas como um evento difícil e traumático, que dificulta a adaptação e a transição saudável. Essas alterações resultam na redefinição dos papéis pessoais, sociais e familiares e na reorganização dos recursos do núcleo familiar. A família movimenta-se de forma a cooperar, organizar e colaborar com as ações de cuidado, especialmente no domicílio, o que favorece a aceitação e adaptação à nova condição de vida^21^. A configuração de uma nova estrutura e dinâmica familiar é uma forma de enfrentar e gerenciar a situação do adoecimento e as demandas advindas com a estomia^22^.

Aceitar o adoecimento e a estomia como consequência terapêutica pode influenciar de forma positiva na qualidade de vida, o que ameniza os desafios que se apresentam cotidianamente^23^. Ainda, as respostas emocionais são fatores importantes para a aceitação e, consequentemente, na autoeficácia para o cuidado e o autocuidado, ao ajudar a superar as mudanças e influenciar na capacidade de reconstituir com sucesso o sentido da vida^24^.

Inicialmente, as pessoas e suas famílias tendem a negar a doença na tentativa de evitar o procedimento cirúrgico, o que resulta em comportamentos disfuncionais no pós-operatório. Estudo realizado com 100 pessoas com estomia intestinal identificou que o pós-operatório é o estágio mais crítico e sofrido, o que compromete o bem-estar e faz emergir a incapacidade de controlar os efeitos colaterais desagradáveis que advêm da estomia, ao exacerbar o sentimento de rejeição, resultando no isolamento social e na demora em aceitar a situação vivida^25^. Viver com uma estomia faz com que a pessoa experiencie emoções extremas e sentimentos negativos que dominam sua vida antes e depois da cirurgia, pois implica em mudanças inevitáveis, sendo concebido como um momento crítico. Essa nova realidade afeta a condição física e psicológica, torna-os vulneráveis, dependentes e altera o estilo de vida, as atividades diárias, com restrições e mudanças significativas no âmbito pessoal e social^5^. Assim, o período transicional confirma as alterações percebidas e vivenciadas por essas pessoas, e envolve alguns acontecimentos críticos que repercutem nas dimensões biopsicossociais, e que modificam diretamente seus estilos de vida.

Dentre os fatores que influenciam o curso da transição, têm-se as particularidades do contexto de vida como facilitadores e/ou inibidores do processo, o que engloba os condicionantes pessoais, como o perfil sociodemográfico e clínico/cirúrgico, o significado emocional, as crenças e a preparação que antecipa conhecimentos e permite elaborar as mudanças que serão vivenciadas.

Em estudo^15^ realizado com o objetivo de conhecer os facilitadores da transição para o autocuidado de pessoas com estomia, identificaram-se os seguintes condicionantes relacionados à pessoa: a capacidade de atribuir significado positivo à estomia; a disponibilidade de orientações sobre os cuidados e as transformações na vida recebidas no preparo pré-operatório; estabilidade psicológica e, o conforto encontrado na fé e na religiosidade.

Sabe-se que a qualidade de vida das pessoas com estomia tem relação com fatores demográficos como idade, estado civil, local da residência e educação^26^. A idade avançada causa maiores dificuldades na vida cotidiana com estomia, e traz dificuldade de cuidar da pele e do equipamento coletor. Para os solteiros, a qualidade de vida se deteriorou rapidamente depois da estomia. O local da residência (rural ou urbana) afeta a qualidade de vida percebida, em especial para quem vive no campo ou em grandes cidades, dadas as dificuldades para cuidar da estomia e da bolsa coletora. Quanto maior o nível de escolaridade, melhor a qualidade de vida percebida após a estomia, sendo que o tempo de estomia também interfere diretamente nesta percepção. No que concerne aos aspectos clínicos e cirúrgicos, têm-se a presença de comorbidades, a localização inadequada da estomia e a presença de complicações pós-operatórias (precoces e tardias) como preditores que inibem o processo de transição, uma vez que dificultam e comprometem a adaptação à nova condição de vida^27^. 

Essas características influenciam também o significado emocional atribuído ao processo de transformação e mudança, os quais têm relação com as crenças individuais e familiares. A espiritualidade e a fé são recursos acessados no intuito de amenizar o sofrimento, trazendo conforto e esperança^28^. Ao dar sentido ao vivido a partir de uma perspectiva neutra ou otimista, essas pessoas conseguem reorganizar-se de forma cooperativa para superar as dificuldades e as limitações, sem dar importância significativa para os inconvenientes, o que direciona suas atitudes e condutas em direção ao bem-estar. Quando os significados e sentimentos são negativos, podem inibir os mecanismos adaptativos e os padrões de resposta, resultando em atitudes disfuncionais que geram instabilidade e vulnerabilidade no núcleo familiar, o que compromete a transição saudável. 

Ante o exposto, é evidente que implementar intervenções de enfermagem que aumentem padrões de respostas positivas, como a autoconfiança, faz com que se sintam responsáveis e envolvidos no processo de transição e na continuação de suas vidas, acessando seus recursos pessoais (competências, autoeficácia) e/ou recursos externos (redes de apoio informais e profissionais)^3^. Para tanto, é relevante que o cuidado transcenda a dimensão biológica com enfoque nas mudanças físicas e fisiológicas, atentando também para os aspectos biopsicossociais, por meio de um plano de cuidados direcionado às suas necessidades e que valorize as mudanças que se apresentam com a nova condição de vida^29^. 

O apoio recebido da família, dos serviços de saúde, da associação de pessoas com estomia, das relações interpessoais, bem como os aspectos relativos à convivência social, constitui-se como recursos externos disponíveis no ambiente comunitário e social, e influenciam a experiência vivida à medida que facilitam ou inibem o processo de transição^24^. Quando a família e as relações conjugais encontram-se fragilizadas, elas podem repercutir negativamente na aceitação e adaptação da pessoa com estomia^9^ ^,^ ^30^. 

O apoio dos serviços de saúde disponíveis na comunidade tem um impacto positivo na vida das pessoas com estomia e da sua família. O suporte do Estado e do Sistema de Saúde possibilita a continuidade do atendimento e da educação após a alta hospitalar, com destaque para as intervenções de cuidado do enfermeiro, que visam contribuir para aliviar os efeitos adversos ocasionados pela estomia, reduzir o estresse e amenizar os problemas, a fim de melhorar a qualidade de vida^5^. 

As intervenções de enfermagem promovem o potencial pessoal e familiar, sendo essenciais para a promoção do autocuidado e da independência. A transição para o autocuidado é um elemento central na experiência da pessoa com estomia no que tange à sua reabilitação. É resultado de um processo complexo de transformação que advém do domínio de novas habilidades e competências, a fim de amenizar os eventos críticos e facilitar o processo de mudança comportamental. Ao facilitar o processo de adaptação, embasado nas especificidades da experiência vivida por essas pessoas, é possível direcionar perspectivas para transições mais salutares, que tenham como desfecho respostas que direcionam a reabilitação para (con)viver com a estomia.

No entanto, é imperativo reconhecer as limitações da teoria, visto que apresenta um escopo e nível de abstração restritos. À medida que a teoria for integrada à prática clínica, sua validação e refinamento contínuos são essenciais para garantir sua eficácia e relevância a longo prazo. A sugestão da necessidade de validação externa não limita a sua aplicabilidade, visto que se torna importante testar e aplicar a teoria em diferentes contextos de cuidado às pessoas com estomia e às suas famílias. Essa validação não apenas fortalecerá a sua estrutura teórica, mas também contribuirá para seu contínuo desenvolvimento. 

Recomenda-se a realização de estudos futuros que se concentrem na aplicação e validação desta teoria na prática clínica, a fim de testá-la em diferentes contextos de cuidado, diretamente com pessoas com estomias intestinais e seus familiares, para avaliar sua efetividade na orientação das intervenções de enfermagem e na promoção de transições saudáveis. A validação externa, tanto por enfermeiros especialistas quanto pela população-alvo, não apenas fortalecerá sua estrutura teórica, mas também contribuirá para seu contínuo desenvolvimento e refinamento.

## Conclusão

Foi possível desenvolver a TSE representativa da experiência de transição vivida pela pessoa com estomia intestinal e sua família. Ao considerar a abordagem integrativa adotada neste estudo, a construção da TSE é uma contribuição significativa para o conhecimento da enfermagem, especialmente no que diz respeito ao cuidado às pessoas com estomia intestinal e suas famílias. Sua estrutura teórica apresenta sete conceitos que descrevem e explicam a experiência de transição vivida por essas pessoas, ao proporcionar uma visão abrangente e holística de um processo complexo e multifacetado, visto que examina não apenas os aspectos individuais, mas também as implicações familiares e sociais. 

Ainda, evidencia que a adaptação à estomia intestinal não se resume às transformações físicas e fisiológicas do corpo, mas resulta em uma reconfiguração da pessoa e da sua identidade, das suas relações e responsabilidades dentro e fora da família. A redefinição de papéis no núcleo familiar, impulsionada por essa transição, destaca a importância de estratégias de intervenção que considerem não apenas a pessoa com estomia, mas também os membros da família como participantes ativos no processo de adaptação. 

É essencial ressaltar que a teoria contribui para fornecer uma base teórica consistente, capaz de sustentar e orientar as etapas do PE, por meio da utilização de linguagem padronizada, auxiliando o raciocínio clínico e a tomada de decisão. Assim, tem potencial de melhorar significativamente o cuidado de enfermagem, pois é embasada em evidências científicas e empíricas que possibilitam organizar um *continuum* de ações preventivas e terapêuticas abrangentes e sensíveis às necessidades dessa população, promovendo uma abordagem efetiva e integral. 

A teoria não só informa a prática, mas também orienta a criação de protocolos operacionais, fluxogramas e outros instrumentos metodológicos que possam auxiliar o cuidado de enfermagem. Essas ferramentas podem contribuir para a sistematização do cuidado, assegurando uma abordagem consistente e qualificada nos diferentes cenários de atenção à saúde, destacando sua aplicabilidade prática.

## Data Availability

Os conjuntos de dados relacionados a este artigo estarão disponíveis mediante solicitação ao autor correspondente.

# Translation: Teoría de situación específica de la experiencia de transición vivida por la persona con ostomía intestinal y su familia*

**DOI:** 10.1590/1518-8345.7979.4857

**Authors:** Angélica Dalmolin, Eduardo da Silva Gomes, Juliano Teixeira Moraes, Leonardo Rozinelli, Maria Ribeiro Lacerda, Nara Marilene Oliveira Girardon-Perlini

**Affiliations:** 1 Universidade Federal de Santa Maria, Enfermagem, Santa Maria, RS, Brazil.; 2 Becaria de la Coordenação de Aperfeiçoamento de Pessoal de Nível Superior (CAPES), Brazil.; 3 Becaria (o) del Conselho Nacional de Desenvolvimento Científico e Tecnológico (CNPq), Brazil.; 4 Universidade Federal de São João del-Rei, Enfermagem, Minas Gerais, MG, Brazil.; 5 Universidade Federal de Santa Maria, Medicina, Santa Maria, RS, Brazil.; 6 Universidade Federal do Paraná, Enfermagem, Curitiba, PR, Brazil.

**Keywords:** Estomía, Familia, Atención de Enfermería, Teoría de Enfermería, Estomaterapia, Modelos de Enfermería

## Abstract

**Objetivo:**

desarrollar una teoría de la situación específica que represente la experiencia de transición vivida por las personas con ostomía intestinal y sus familias, basada en la Teoría de la Transición de la Meleis Afaf.

**Método:**

estudio teórico, guiado por la estrategia teoría-investigación-teoría y basado en el enfoque integrativo, que combina estrategias deductivas e inductivas. En la etapa deductiva, se enumeró la Teoría de las Transiciones para proporcionar el marco conceptual y organizar el pensamiento teórico. Las estrategias inductivas implicaron una revisión integradora, análisis documental y el desarrollo de un grupo focal.

**Resultados:**

Se desarrolló una teoría específica para cada situación que describe y explica la experiencia de transición vivida por las personas con ostomía intestinal y su familia a través de siete conceptos y 20 subconceptos. El marco teórico también abarca enunciados relacionales, así como un diagrama representativo de sus conceptos y sus interrelaciones.

**Conclusión:**

la teoría desarrollada tiene el potencial de guiar y sistematizar la atención de enfermería para personas con ostomía y sus familias, considerando las diferentes fases del proceso de transición.

## Destacados

**(1)** La TSE es una contribución significativa al conocimiento de la enfermería. **(2)** Incluye aspectos individuales y las implicaciones familiares y sociales de la transición. **(3)** Tiene potencial para guiar y sistematizar la atención durante la transición. **(4)** Es capaz de sostener y orientar las etapas del Proceso de Enfermería. **(5)** Se puede aplicar a los diferentes escenarios de atención a la salud.

## Introducción

La enfermedad provoca rupturas en la relación de los seres humanos con su mundo cotidiano, siendo un acontecimiento impredecible y significativo dado el impacto en la vida personal, familiar y comunitaria^1^. Puede ir acompañado de diferentes grados de dependencia física y múltiples sentimientos, lo que resulta en cambios significativos que retrasan planes y proyecciones de futuro.

La imprevisibilidad de los hechos derivados de la enfermedad altera el estado del equilibrio individual y familiar, lo que provoca la necesidad de una reorganización en vista de la transición experimentada^1^. La transición es el paso de una fase de la vida, condición o estatus a otra, siendo el resultado de complejas interacciones entre las personas y el medio ambiente. Consiste en pasar de un estado estable a otro estado relativamente estable^2^.

Estar enfermo representa una transición en la vida de las personas que se caracteriza como una experiencia única, multidimensional y compleja, asociada a periodos de inestabilidad emocional, tanto en la vida individual como familiar, generando reacciones y comportamientos ante la experiencia de la enfermedad^2^. Esto lleva a la persona y/o a su familia a atravesar diferentes fases, pasando de una condición de comodidad, seguridad y tranquilidad a periodos de desequilibrio, incertidumbres y conflictos, lo que genera cambios de comportamiento para encontrar estabilidad^3^ ^-^ ^4^.

El diagnóstico de una enfermedad, acompañado de la preparación de una ostomía intestinal, representa un proceso de transición notable, generando una ambivalencia de sentimientos que resulta en incertidumbres, culminando en la necesidad de nuevas estrategias de afrontamiento para restablecer el equilibrio y el bienestar^5^. Esta realidad implica cambios en el entorno familiar, ya que las situaciones de cuidado impuestas por la enfermedad y la presencia de la ostomía reflejan en los demás miembros de la familia e impactan en la dinámica familiar, caracterizándolo como un evento colectivo^6^ ^-^ ^8^.

La familia es el núcleo principal de las relaciones interpersonales para las personas con ostomía y ejerce una influencia significativa en la rehabilitación a través de su coparticipación en acciones de cuidado^9^. El apoyo familiar es un factor determinante para la experiencia de transición y puede reflejar positiva o negativamente en el proceso de aceptación y adaptación a la nueva condición vital^10^. La construcción de la ostomía despierta nuevas y específicas necesidades de cuidado, promoviendo cambios físicos y fisiológicos, afectando también las dimensiones psicosociales y espirituales^11^ ^-^ ^12^. 

La ostomía pone a la luz las vulnerabilidades y fragilidades de la persona y la familia, que se manifiestan en implicaciones de trastornos organizativos, emocionales y psicológicos, como el deterioro de habilidades y capacidades para las tareas diarias, la distorsión de la autoimagen por mutilación del cuerpo, el miedo y la inseguridad que pueden conducir al aislamiento social. Por tanto, es necesario que la atención de enfermería implique atención en medio de cambios que trascienden las dimensiones físicas y fisiológicas, entrando en los aspectos subjetivos que acompañan al proceso de transición experimentado. En vista de esto, las enfermeras pueden entretejer estrategias de atención para contribuir significativamente a la construcción de una experiencia y experiencia positiva^13^ ^-^ ^15^.

Teniendo en cuenta la complejidad que implica la atención de enfermería para estas personas y sus familias, conviene destacar la necesidad de profundizar en el conocimiento teórico como alternativa para guiar y calificar la atención. El desarrollo de teorías representativas de la experiencia de transición puede contribuir a caracterizar los fenómenos de interés para los profesionales en el cuidado de esta población, apoyando la identificación de respuestas humanas y la implementación de intervenciones basadas en el Proceso de Enfermería. Los marcos teóricos que describen o explican esta experiencia de transición contribuyen al paradigma de la práctica guiada por la teoría y a la organización y sistematización del conocimiento científico y filosófico en enfermería^16^ ^-^ ^17^.

En este contexto, las teorías de situaciones específicas (TSE) destacan por su capacidad de representar y describir un conjunto limitado de conceptos, explicando la relación entre ellos, para elaborar una predicción de los resultados de esta interrelación. Así pues, son más específicas, diferenciándose de otras teorías por su nivel de abstracción, alcance y contexto^18^.

La elaboración de una TSE permite una visión sensible de la atención de enfermería, considerando las múltiples dimensiones que abarcan el proceso de transición que experimenta la persona con ostomía y su familia, con el objetivo de organizar una atención integral, singular e integral, haciendo posible planificar acciones específicas que sean congruentes con las necesidades de esta población. En vista de lo anterior, este estudio se basó en la siguiente pregunta de investigación: ¿qué elementos constituyentes forman parte de una teoría de situación específica que representa la experiencia de transición vivida por las personas con ostomía intestinal y sus familias? El objetivo de este estudio fue desarrollar una TSE representativa de la experiencia de transición vivida por personas con ostomía intestinal y sus familias, considerando la Teoría de la Transición (TT) de la Afaf Meleis.

## Método

### Diseño del estudio

Se trata de un estudio teórico, guiado por la estrategia teoría-investigación-teoría y basado en el enfoque integrador para el desarrollo de una TSE, de tipo descriptivo y explicativo^19^.

### Recogida de datos

Se emplearon estrategias deductivas e inductivas para el desarrollo de la TSE. En la fase deductiva, se incluyó la TT de Afaf Meleis para proporcionar el marco conceptual y organizar el pensamiento teórico. Las estrategias inductivas incluyeron el desarrollo de una revisión integradora de la literatura, análisis documental y una investigación cualitativa, de naturaleza descriptivo-exploratoria.

### Estrategia deductiva

La TT proporcionó la base de analogía para las explicaciones y entendimientos sobre el fenómeno de interés. Así, la aproximación con el marco teórico se llevó a cabo mediante una lectura minuciosa y cuidadosa, lo que permitió extraer conceptos y subconceptos de la TT que pudieran contribuir a la representación de la experiencia de transición vivida por la persona con ostomía intestinal y su familia. Los conceptos deducidos de la TT para el desarrollo de la TSE fueron: tipos y patrones de transición; propiedades de experiencia en transición; condiciones de transición (facilitantes e inhibidoras); indicadores de proceso; indicadores de resultados; y terapia de enfermería. Los conceptos derivados de la TT constituyeron la base para el análisis de la literatura científica mediante revisión integradora y análisis documental. Así, se crearon 28 códigos correspondientes a cada concepto y subconcepto derivado de la TT en el *software* Atlas.ti 22, para facilitar la recogida de datos en las etapas siguientes. El marco teórico de TT también proporcionó posibles enunciados relacionales entre los conceptos que surgieron durante el proceso de teorización. 

### Estrategia inductiva: revisión integradora de la literatura

La revisión integradora buscó ampliar la comprensión de la transición que experimentan las personas con ostomía y sus familias mediante el análisis de evidencia científica. Se operacionalizó considerando seis etapas predefinidas, a saber: formulación de la pregunta de revisión; muestras de literatura; categorización de estudios; evaluación de los estudios incluidos; interpretación de resultados y síntesis de conocimientos. 

Inicialmente, se definieron el tema y el problema de la investigación, lo que permitió elaborar la siguiente pregunta de revisión: “¿Cuáles son las experiencias de las personas con ostomía intestinal y sus familias en relación con enfermedades y formación de ostomías?”.

Posteriormente, se realizaron búsquedas en las siguientes bases de datos: Literatura Latinoamericana y Caribeña sobre Ciencias de la Salud (LILACS); Base de Datos Brasileña de Enfermería (BDENF); Índice Bibliográfico Español en Ciencias de la Salud (IBECS); Sistema de Análisis y Recuperación de Literatura Médica Online (MEDLINE)/Biblioteca Nacional de Medicina de Estados Unidos (PubMed); Índice Acumulativo de Literatura de Enfermería y Salud Afines (CINAHL); y *Web of Science*. Se emplearon diferentes estrategias de búsqueda en cada base de datos, combinando distintos descriptores y palabras clave con operadores booleanos, como se detalla a continuación:

Primera estrategia: (estoma OR estomia OR ostoma OR ostomia OR “estomas cirúrgicos” OR colostomia OR ileostomia OR estomaterapia OR enterostomia) AND (família OR famílias OR familiares OR familiar OR “membros da família”) AND (experiência OR experiências OR vivência OR vivências OR impacto OR repercussão OR repercussões OR adaptação OR adaptações OR implicação OR implicações OR sentimentos OR emoções OR sexualidade OR aceitação OR reabilitação OR “qualidade de vida”). 

Segunda estrategia: ((ostomy OR colostomy OR ileostomy OR “surgical stomas”) AND (family OR families) AND (“life change events” OR experience OR experiences OR recovering OR adaptation OR implication OR feeling OR emotions OR sexuality OR acceptance OR “quality of life”)).

Se incluyeron estudios en inglés, portugués o español, disponibles íntegramente y cuyo objeto de investigación se basaban en las experiencias de las personas y sus familias ante la enfermedad y la preparación de la ostomía intestinal. Los criterios de exclusión fueron: estudios realizados con niños y adolescentes, o incluso aquellos que abordaran otros tipos de ostomía, distintos a los intestinales, además de tesis, tesis, monografías, manuales, capítulos de libros, informes de experiencia, reflexiones, revisiones y editoriales.

De las búsquedas se obtuvieron 1.490 estudios, que se importaron al *software* EndNote Basic. De este total, 309 fueron excluidos por duplicarse, lo que resultó en 1.181 publicaciones. El análisis de estas referencias se operacionalizó en el *software* Rayyan, en el que inicialmente se leyeron los títulos y resúmenes, lo que resultó en la exclusión de 1.094 estudios por no cumplir los criterios establecidos. Así, se preseleccionaron 87 estudios y se leyeron en su totalidad. De una lectura exhaustiva, se encontró que 48 estudios no respondieron a la pregunta de la revisión, lo que permitió delimitar el corpus de análisis en 39 artículos. La encuesta bibliográfica se llevó a cabo entre marzo y junio de 2022 a través del Portal de la Revista CAPES, de forma doble independiente. Cuando hubo desacuerdos sobre la elegibilidad de las publicaciones, estos se resolvieron mediante consenso y análisis de un tercer revisor. Es importante destacar que el proceso de selección y análisis de los artículos siguió las recomendaciones del protocolo *Preferred Reporting Items for Systematic Reviews and Meta-Analysis* (PRISMA).

La interpretación de los datos se llevó a cabo a la luz de la TT, siguiendo el razonamiento inductivo-deductivo, con el fin de identificar la evidencia relacionada con los conceptos de la teoría, considerando el fenómeno en cuestión. Con el análisis, se crearon citas, es decir, segmentos del texto considerados relevantes y que correspondían a los códigos previamente establecidos durante la estrategia deductiva, así como citas libres, que posteriormente se asociaron para crear nuevos códigos. 

Esta etapa permitió la conjugación del razonamiento inductivo en el proceso de teorización y la formación de dos nuevos conceptos y 11 subconceptos, basados en la evidencia identificada en la literatura. Así, al final de esta etapa, se amplió la estructura de la emergente TSE, conformando ocho conceptos y 33 subconceptos, lo que permitió organizar una estructura teórica capaz de representar las experiencias de las personas y sus familias en relación con la enfermedad y la formación de la ostomía.

### Estrategia inductiva: análisis documental

En esta etapa, se analizaron las producciones científicas relacionadas con el tema de la atención de enfermería para personas con ostomía intestinal y sus familias del Núcleo de Estudios en Cuidado y Familia (NECFAM) de la Universidad Federal de Santa María. Las producciones de NECFAM sobre ostomías intestinales fueron una fuente de datos para el desarrollo de la ETS, complementando el proceso integrador de teorías. 

Se analizaron 13 documentos, de investigación e informes de extensión; monografías de finalización de graduación; Tesis de máster y tesis doctoral. El análisis documental buscó responder a las siguientes preguntas de revisión: “¿Cuáles son las experiencias de las personas con ostomía intestinal y sus familias en relación con la enfermedad y la formación de ostomías?”, y “¿Cuáles son las acciones de cuidado de enfermería con personas con ostomía intestinal y sus familias?”. 

Inicialmente, este paso se operacionalizó mediante la extracción de datos para tablas sinópticas, que se exportaron al *software* Atlas.ti 22. Posteriormente, el análisis y la síntesis de la evidencia se realizaron mediante lecturas interpretativas a la luz de la TT, siguiendo el razonamiento inductivo-deductivo. A partir de esto, se elaboraron nuevas citas, destacando en los documentos segmentos del texto considerados relevantes y que correspondían a los códigos que se referían a los conceptos y subconceptos de la TT, así como citas libres, que posteriormente se asociaron con nuevos códigos.

La síntesis del análisis documental permitió vincular los códigos libres a los grupos de códigos relacionados con los conceptos y subconceptos de la TT, así como a los nuevos conceptos y subconceptos identificados en el paso anterior. Así, la estructura teórica desarrollada hasta la etapa de revisión integradora no se amplió, pero la evidencia identificada en el análisis documental proporcionó una mayor densidad teórica a los conceptos previamente elaborados, además de clarificar nuevos aspectos relacionados con las experiencias de las personas y sus familias en relación con la enfermedad y la realización de la ostomía. Al final de esta etapa, fue posible establecer y confirmar las relaciones entre los conceptos de la TSE mediante un proceso de abstracción que consideró la evidencia científica y la estructura teórica de la TT. Así, se elaboró un diagrama representativo de la teoría emergente, señalando los conceptos, subconceptos y sus relaciones, así como las fases que componen el proceso de transición experimentado por la persona con ostomía y su familia.

### Estrategia inductiva: grupo focal

Para fortalecer, ampliar, refinar o refutar los conceptos y afirmaciones elaborados a partir de la deducción de la TT y la inducción, a través de los hallazgos de la revisión bibliográfica y el análisis documental, se desarrolló un estudio cualitativo de naturaleza descriptivo-exploratoria, utilizando la técnica de grupo focal (GF). Los participantes estaban formados por seis miembros del NECFAM por sus experiencias previas en investigación, docencia y extensión sobre el tema de la atención de enfermería para personas con ostomía intestinal y sus familias. La selección de los participantes se realizó intencionadamente y la muestra se consideró adecuada para refinar la estructura teórica desarrollada, en la que la profundidad de la discusión y la experiencia de los participantes son más relevantes que la búsqueda de saturación de nuevos temas, lo cual está en línea con las directrices metodológicas para la operacionalización de GF.

Los criterios de selección fueron: ser miembro de NECFAM, tener al menos seis meses de participación en proyectos sobre el tema y tener una publicación científica en el área. Se excluía a los miembros que no conocían el tema o que no frecuentaban las reuniones asiduamente.

De los seis participantes, dos eran doctores, dos eran estudiantes de posgrado, uno a nivel doctoral y otro a nivel de máster, y dos eran estudiantes de grado. Para operacionalizar el GF, se preparó una presentación de la TSE en un recurso digital en Microsoft PowerPoint y los participantes recibieron previamente la síntesis de la estructura teórica (presentación de la teoría) en un documento de texto en Microsoft Word. Además, se preparó una guía de temas para mediar las discusiones del grupo basadas en los conceptos, subconceptos, enunciados relacionales y el diagrama teórico, que también compuso el material organizado para la presentación y la síntesis previamente enviada. La guía abordaba la comprensión del proceso de transición, la naturaleza y propiedades de los conceptos, los factores condicionantes de la transición, así como los patrones de respuesta y su aplicabilidad en la práctica clínica.

Las reuniones del GF se operacionalizaron en tres momentos: recepción e integración entre los participantes, recogida de datos del material de apoyo e interacción grupal, síntesis y consenso de lo que se debatió. Se celebraron tres reuniones del GF en fechas diferentes, que tuvieron lugar entre julio y octubre de 2023, y se realizaron por videoconferencia, con la ayuda de la plataforma digital Google *Meet*, que fueron grabadas en audio y vídeo, transcritas íntegramente y complementadas con un diario de campo.

En la primera reunión se discutió la estructura general (conceptos, subconceptos y sus relaciones); en el segundo, se perfeccionó el diagrama representativo; y en el tercero, se validó el diagrama final. A partir de las reuniones del GF, fue posible calificar y refinar la estructura teórica y el diagrama representativo de la teoría, lo que aportó diferentes puntos de vista al fenómeno estudiado. Además, la validez de las contribuciones de los participantes surgió mediante consenso y deliberaciones continuamente afirmadas por el grupo, que sirvieron como el principal mecanismo para garantizar la fiabilidad de dichas contribuciones. Este paso siguió las recomendaciones de los *Consolidated Criteria for Reporting Qualitative Research* (COREQ).

### Aspectos éticos

Para desarrollar este estudio, se respetaron los preceptos éticos de la Resolución n.º 510, del 7 de abril de 2016, del Consejo Nacional de Salud, que trata de las directrices y normas regulatorias para la investigación con seres humanos. Este estudio fue aprobado por el Comité de Ética en la Investigación bajo el número de opinión 5.346.695. 

## Resultados

La integración de los resultados de las estrategias deductiva e inductiva culminó en la organización de siete conceptos y veinte subconceptos para describir y explicar la experiencia de transición de las personas con ostomía intestinal y sus familias, como se muestra en la Figura 1.

**Figura 1:** Conceptos y subconceptos que conforman la TSE* de la experiencia de transición de las personas con ostomía intestinal y sus familias. Santa Maria, RS, Brasil, 2024

Concepto	Subconcepto
Perciben los cambios en la funcionalidad del cuerpo	Identifican y observan los signos y síntomas;
	Incentivan a buscar ayuda;
	Buscan acelerar el diagnóstico.
Niegan la enfermedad y la ostomía	Posponen la búsqueda de atención;
	Posponen la realización de la cirugía;
	Evitan mirar el cuerpo tras la ostomía.
Experimentan tipos y patrones de transición	Confirman el diagnóstico y enfrentan lo desconocido;
	Redefinen los roles y la organización familiar ante el diagnóstico y la ostomía.
Propiedades de la transición	Aceptan la nueva realidad y se involucran colectivamente en el cuidado;
	Confirman eventos críticos y cambios a lo largo del tiempo.
Particularidades del contexto vital como facilitadores y/o inhibidores	Perfil sociodemográfico y clínico/quirúrgico;
	Significado emocional atribuido al vivido con la enfermedad y la ostomía;
	Creencias individuales y familiares en la experiencia vivida;
	Anticipan los conocimientos.
Condicionantes externos como facilitadores y/o inhibidores	Apoyo recibido en los servicios de salud y en la asociación de personas con ostomía;
	La familia en el contexto de transición;
	Relaciones interpersonales de afecto;
	Aspectos relacionados con la convivencia social.
Respuestas que dirijan la rehabilitación a (con)vivir con la ostomía	Cooperación en la búsqueda de estabilidad personal y familiar;
	Dominio de habilidades y comportamientos para la reformulación de la identidad.

*TSE = Teoría de Situación Específica

*Perciben los cambios en la funcionalidad del cuerpo* comprende la fase que precede al proceso de transición, el momento en que se identifican los primeros cambios en la funcionalidad del cuerpo a través de los signos y síntomas*.* Así, existe una movilización intrafamiliar que resulta en un *incentivo para buscar ayuda* en los servicios de salud y *buscar agilizar el diagnóstico*, lo que resulta en un periodo impregnado de ansiedad y miedo.

*Niegan la enfermedad y la ostomía* implica posponer *la búsqueda de atención*, por miedo a confirmar la enfermedad. Ante el diagnóstico no deseado, rechazan la enfermedad en un intento de posponer la cirugía, ya que existe inseguridad e incertidumbre respecto a la preparación de la ostomía. Esto implica comportamientos disfuncionales en el periodo postoperatorio, *como evitar mirar el cuerpo tras la ostomía*, lo que acaba restringiendo la capacidad de la persona y su familia para adaptarse, ya que aún no son conscientes de los procesos de transformación, lo que indica que la transición aún no ha comenzado. 

*Experimentan tipos y patrones de transición* y están relacionados con la *confirmación de la enfermedad por el diagnóstico*, lo que resulta en *afrontar lo desconocido*. El resultado terapéutico es la formación de la ostomía, que se presenta de forma inesperada y genera la necesidad de *redefinir los roles y, al mismo tiempo, organizar los recursos intrafamiliares*. Así, se entiende que estas personas experimentan múltiples transiciones relacionales que se originan en una naturaleza de salud y enfermedad, siendo una experiencia colectiva, con aspectos específicos tanto para la persona como para la familia, pero que están mutuamente influenciadas. 

*Las propiedades de la transición* comprenden la conciencia y percepción de las transformaciones experimentadas, lo que resulta en la *aceptación de la nueva realidad y, por tanto, hace que la persona y su familia se involucren en el cuidado*, mediante la búsqueda de recursos y estrategias que fortalezcan el núcleo familiar. Incluye comportamientos relacionados con la transición, que no son estáticos ni definidos, sino consecuencia de interacciones complejas entre las personas y el entorno. Durante este proceso, *se experimentan cambios y eventos críticos* con el tiempo, lo que genera repercusiones e implica reajustes en varios aspectos y atraviesa un periodo indefinido de inestabilidad y vulnerabilidad. 

*Las particularidades del contexto vital como facilitadores y/o inhibidores* implican *características sociodemográficas y clínicas/quirúrgicas*, así como *la capacidad de establecer un significado emocional a la experiencia*, ser influenciada por creencias individuales y familiares y la preparación necesaria al anticipar el conocimiento para experimentar el proceso de transición, lo que puede limitar o ampliar sus recursos terapéuticos.

*Los factores condicionantes externos, como facilitadores y/o inhibidores*, están relacionados con las condiciones y factores de la comunidad y el entorno social que interfieren directamente con la experiencia vivida. Se refiere al *apoyo recibido en los servicios de salud y en la asociación de personas con ostomías*, que es un recurso que promueve condiciones favorables para las nuevas condiciones de vida. También incluye la *implicación de la familia en el contexto de la transición*, así como *las relaciones interpersonales de afecto*, que pueden facilitar e inhibir las condiciones de este proceso. Los *aspectos relacionados con la convivencia social* se ven directamente afectados en detrimento de las exigencias del cuidado de la ostomía, lo que puede exponerlos a situaciones delicadas que acaban impidiéndoles cumplir con las expectativas sociales. 

*Las respuestas que dirigen la rehabilitación a (con)convivir con la ostomía* implican el proceso experimentado durante el tiempo de transición, siendo la *cooperación y la interacción esenciales en la búsqueda de estabilidad y bienestar personal y familiar*. Esto requiere dominar *nuevas habilidades, competencias y comportamientos*, con el objetivo de *reestructurar una nueva identidad* que permita la continuidad de la vida, basada en la sensación de normalidad y que reconozca los cambios derivados de la transición experimentada. 

### Conceptos y sus afirmaciones relacionales

La síntesis de los resultados permitió establecer la selección de conceptos y subconceptos y elaborar enunciados teóricos relacionales, basados en la indicación de las relaciones entre dos o más conceptos, que culminaron en la creación de un diagrama representativo de la estructura teórica y sus enunciados, como se muestra en la Figura 2. 

*Perciben los cambios en la funcionalidad del cuerpo* está directamente relacionado con experimentan tipos y patrones de transición, ya que conscientemente precede al inicio de la experiencia vivida y está influido por las particularidades del contexto vital, así como por los condicionantes externos que pueden facilitar o inhibir la transición. Es una condición que permite establecer una oposición con niegan la enfermedad y la ostomía, ya que, al reconocer los cambios en el patrón funcional del cuerpo, a menudo pueden negar lo que se está experimentando.

*Niegan la enfermedad y la ostomía* está influida por las particularidades del contexto vital y por los factores de condicionamiento externos que influyen en la experiencia, lo que puede (hacer imposible experimentar tipos y patrones de transición y, por tanto, mantener a la persona con ostomía y a su familia en una fase previa a la transición, o bien, dirigir acciones y respuestas que hacen posible experimentar la transición, dejando el ciclo de negación con cierto grado de conciencia sobre los cambios relacionados con este proceso. 

**Figura 2:** Diagrama representativo de los conceptos y afirmaciones relacionales de la TSE* de la experiencia de transición de las personas con ostomía intestinal y sus familias. Santa Maria, RS, Brasil, 2024

*TSE = Teoría de Situación Específica

*Experimentan tipos y patrones de transición* se correlacionan con las propiedades de la transición, que comprenden características esenciales que indican conciencia y aceptación de los cambios, lo que genera implicación y confirma eventos críticos a lo largo del proceso, con interferencia de las particularidades del contexto vital y las restricciones externas. Este concepto también modula las respuestas que dirigen la rehabilitación a (co)vivir con la ostomía, ya que permite la reorganización intrafamiliar y la redefinición de roles, para restablecer la estabilidad y dominar la nueva realidad de la vida. 

Las *propiedades de la transición* ejercen influencia, especialmente en la fase de transición, que no es lineal ni estática, sino el producto de interacciones complejas de la persona consigo misma, con su familia y con el entorno. Es un reflejo de la conciencia sobre lo que se está viviendo, lo que tiene repercusiones en su nivel de implicación y, por tanto, inspira comportamientos, reacciones y respuestas que dirigen la rehabilitación. También se ven interfiridos por las particularidades del contexto vital y las restricciones externas, que son recursos que pueden facilitar o restringir el proceso hacia una transición saludable. 

Las *particularidades del contexto vital como facilitadores y/o inhibidores* implican especificidades individuales y familiares relacionadas con la experiencia y están relacionadas con factores externos de condicionamiento, que influyen en las condiciones bajo las cuales ocurre la transición. Por tanto, son elementos que están presentes y resuenan significativamente a lo largo de la experiencia de transición, ya que influyen en cómo se mueven estas personas a lo largo del proceso. 

Las *respuestas que dirigen a la rehabilitación a (co)convivir con la ostomía* están relacionadas con tipos de experiencia y patrones de transición, ya que implican enfrentarse a lo desconocido con la confirmación del diagnóstico y la consiguiente redefinición de los roles personales, familiares y sociales. También están relacionadas con las propiedades de la transición, influenciadas por las particularidades del contexto vital y por los condicionantes externos. 

## Discusión

En este estudio, la experiencia de transición de las personas con ostomía y sus familias se anticipó por los primeros signos de enfermedad, que se produjeron a través de la percepción y reconocimiento de los primeros signos y síntomas. A partir de esto, surge una movilización interna que da lugar a la búsqueda de ayuda en diferentes servicios de salud, públicos y/o privados, como forma de acelerar el diagnóstico. Así, también se evidencia el itinerario terapéutico de las personas con ostomía en la búsqueda de atención especializada, comenzando con el descubrimiento de alteraciones físicas, seguido del acceso a servicios de salud como forma de formalizar el diagnóstico y buscar tratamiento^20^.

Para las familias, el origen de la experiencia de convivir con un familiar adulto con una ostomía debido a un cáncer intestinal proviene de la interacción con el familiar enfermo, cuando se dan cuenta de que algo no va bien, observando signos de la enfermedad en la manifestación de cambios funcionales en el intestino. Las familias comprenden que es necesario buscar atención en los servicios de salud y animar a sus familiares a obtener respuestas sobre los signos y síntomas presentados^8^.

En la experiencia de transición, la enfermedad y la ostomía aparecen de forma inesperada, percibidas como un evento difícil y traumático, que dificulta la adaptación y una transición saludable. Estos cambios resultan en la redefinición de los roles personales, sociales y familiares y en la reorganización de los recursos del núcleo familiar. La familia se traslada para cooperar, organizar y colaborar con acciones de cuidado, especialmente en casa, lo que favorece la aceptación y adaptación a las nuevas condiciones de vida^21^. La configuración de una nueva estructura y dinámica familiar es una forma de afrontar y gestionar la situación de enfermedad y las demandas derivadas de la ostomía^22^.

Aceptar la enfermedad y la ostomía como consecuencia terapéutica puede influir positivamente en la calidad de vida, lo que mitiga los desafíos que se presentan a diario^23^. Además, las respuestas emocionales son factores importantes para la aceptación y, en consecuencia, para la autoeficacia en el cuidado y el autocuidado, ya que ayudan a superar cambios e influyen en la capacidad para reconstituir con éxito el sentido de la vida^24^.

Inicialmente, las personas y sus familias tienden a negar la enfermedad para intentar evitar la intervención quirúrgica, lo que provoca conductas disfuncionales en el periodo postoperatorio. Un estudio realizado con 100 personas con ostomía intestinal identificó que el periodo postoperatorio es la etapa más crítica y dolorosa, lo que compromete el bienestar y pone de manifiesto la incapacidad para controlar los efectos secundarios desagradables derivados de la ostomía, agravando la sensación de rechazo, lo que provoca aislamiento social y retraso en aceptar la situación experimentada^25^. Vivir con una ostomía provoca que la persona experimente emociones extremas y sentimientos negativos que dominan su vida antes y después de la cirugía, ya que implica cambios inevitables, siendo concebidos como un momento crítico. Esta nueva realidad afecta su condición física y psicológica, los vuelve vulnerables, dependientes y altera su estilo de vida, sus actividades diarias, con restricciones y cambios significativos en las esferas personal y social^5^. Así, el periodo de transición confirma los cambios percibidos y experimentados por estas personas, e implica algunos eventos críticos que tienen repercusiones en las dimensiones biopsicosociales y que modifican directamente sus estilos de vida.

Entre los factores que influyen en el curso de la transición están las particularidades del contexto vital como facilitadores y/o inhibidores del proceso, que abarcan factores de condicionamiento personal, como el perfil sociodemográfico y clínico/quirúrgico, el significado emocional, las creencias y la preparación que anticipa el conocimiento y permite la elaboración de los cambios que se experimentarán.

En un estudio^15^ realizado con el objetivo de conocer a los facilitadores de la transición al autocuidado de las personas con ostomía, se identificaron los siguientes factores de condicionamiento relacionados con la persona: la capacidad de atribuir un significado positivo a la ostomía; la disponibilidad de orientación sobre cuidados y transformaciones vitales recibidas en la preparación preoperatoria; estabilidad psicológica y el consuelo que se encuentra en la fe y la religiosidad.

Se sabe que la calidad de vida de las personas con ostomía está relacionada con factores demográficos como la edad, el estado civil, el lugar de residencia y la educación^26^. La edad avanzada causa mayores dificultades en la vida diaria con la ostomía, y dificulta el cuidado de la piel y el equipo de recogida. Para los solteros, la calidad de vida se deterioró rápidamente tras la ostomía. El lugar de residencia (rural o urbano) afecta a la calidad de vida percibida, especialmente para quienes viven en el campo o en grandes ciudades, dadas las dificultades para cuidar la ostomía y la bolsa colectora. Cuanto mayor es el nivel educativo, mejor es la calidad de vida percibida tras la ostomía, y el momento de la ostomía también interfiere directamente con esta percepción. En cuanto a los aspectos clínicos y quirúrgicos, la presencia de comorbilidades, la localización inadecuada de la ostomía y la presencia de complicaciones postoperatorias (tempranas y tardías) son predictores que inhiben el proceso de transición, ya que dificultan y comprometen la adaptación a la nueva condición vital^27^. 

Estas características también influyen en el significado emocional atribuido al proceso de transformación y cambio, que están relacionados con las creencias individuales y familiares. La espiritualidad y la fe son recursos a los que se accede con el objetivo de aliviar el sufrimiento, aportar consuelo y esperanza^28^. Al dar sentido a lo que han experimentado desde una perspectiva neutral u optimista, estas personas son capaces de reorganizarse de forma cooperativa para superar dificultades y limitaciones, sin dar una importancia significativa a las molestias, lo que orienta sus actitudes y comportamientos hacia el bienestar. Cuando los significados y sentimientos son negativos, pueden inhibir mecanismos adaptativos y patrones de respuesta, lo que resulta en actitudes disfuncionales que generan inestabilidad y vulnerabilidad en el núcleo familiar, comprometiendo la transición saludable. 

A la vista de lo anterior, es evidente que implementar intervenciones de enfermería que aumenten patrones de respuesta positiva, como la autoconfianza, les hace sentirse responsables e implicados en el proceso de transición y en la continuidad de sus vidas, accediendo a sus recursos personales (competencias, autoeficacia) y/o recursos externos (redes de apoyo informales y profesionales)^3^. Por lo tanto, es relevante que el cuidado trascienda la dimensión biológica con un enfoque en los cambios físicos y fisiológicos, prestando también atención a los aspectos biopsicosociales, a través de un plan de cuidados orientado a sus necesidades y que valore los cambios que se presentan con la nueva condición de vida^29^. 

El apoyo recibido de la familia, los servicios de salud, la asociación de personas con ostomías, las relaciones interpersonales, así como aspectos relacionados con la convivencia social, constituyen recursos externos disponibles en la comunidad y el entorno social, e influyen en la experiencia vivida al facilitar o inhibir el proceso de transición^24^. Cuando las relaciones familiares y matrimoniales se debilitan, pueden tener un impacto negativo en la aceptación y adaptación de la persona con ostomía^9^ ^,^ ^30^. 

El apoyo a los servicios de salud disponibles en la comunidad tiene un impacto positivo en la vida de las personas con ostomía y sus familias. El apoyo del Estado y del Sistema Sanitario permite la continuidad de la atención y la educación tras el alta hospitalaria, con énfasis en las intervenciones de atención de las enfermeras, que buscan contribuir a aliviar los efectos adversos causados por la ostomía, reducir el estrés y aliviar los problemas, con el fin de mejorar la calidad de vida^5^. 

Las intervenciones de enfermería promueven el potencial personal y familiar, y son esenciales para fomentar el autocuidado y la independencia. La transición al autocuidado es un elemento central en la experiencia de las personas con ostomía respecto a su rehabilitación. Es el resultado de un proceso complejo de transformación que surge del dominio de nuevas habilidades y competencias, con el fin de mitigar eventos críticos y facilitar el proceso de cambio de comportamiento. Al facilitar el proceso de adaptación, basado en las especificidades de la experiencia vivida por estas personas, es posible orientar perspectivas para transiciones más saludables, que tienen como resultado respuestas que dirigen la rehabilitación para (con)vivir con la ostomía.

Sin embargo, es imprescindible reconocer las limitaciones de la teoría, dado que presenta un alcance y nivel de abstracción restringidos. A medida que la teoría se integra en la práctica clínica, su validación y perfeccionamiento continuos son esenciales para garantizar su eficacia y relevancia a largo plazo. La sugerencia de la necesidad de validación externa no limita su aplicabilidad, ya que es importante probar y aplicar la teoría en diferentes contextos de atención para personas con ostomía y sus familias. Esta validación no solo fortalecerá su marco teórico, sino que también contribuirá a su desarrollo continuo. 

Se recomienda realizar futuros estudios centrados en la aplicación y validación de esta teoría en la práctica clínica, con el fin de probarla en diferentes contextos de atención, directamente con personas con ostomías intestinales y sus familias, para evaluar su eficacia en la orientación de las intervenciones de enfermería y la promoción de transiciones saludables. La validación externa, tanto por parte de enfermeros especialistas como de la población objetivo, no solo fortalecerá su marco teórico, sino que también contribuirá a su desarrollo y perfeccionamiento continuos.

## Conclusión

Fue posible desarrollar el representante de la TSE de la experiencia de transición vivida por la persona con ostomía intestinal y su familia. Teniendo en cuenta el enfoque integrador adoptado en este estudio, la construcción de la TSE supone una contribución significativa al conocimiento de enfermería, especialmente en lo que respecta al cuidado de personas con ostomía intestinal y sus familias. Su marco teórico presenta siete conceptos que describen y explican la experiencia de transición vivida por estas personas, proporcionando una visión integral y holística de un proceso complejo y multifacético, ya que examina no solo aspectos individuales, sino también implicaciones familiares y sociales. 

También muestra que la adaptación a la ostomía intestinal no se limita a las transformaciones físicas y fisiológicas del cuerpo, sino que resulta en una reconfiguración de la persona y su identidad, sus relaciones y responsabilidades dentro y fuera de la familia. La redefinición de los roles en el núcleo familiar, impulsada por esta transición, pone de manifiesto la importancia de estrategias de intervención que consideren no solo a la persona con ostomía, sino también a los miembros de la familia como participantes activos en el proceso de adaptación. 

Es fundamental enfatizar que la teoría contribuye a proporcionar una base teórica coherente, capaz de sostener y guiar las etapas del PE, mediante el uso de lenguaje estandarizado, facilitando el razonamiento clínico y la toma de decisiones. Por tanto, tiene el potencial de mejorar significativamente la atención de enfermería, ya que se basa en evidencia científica y empírica que permite organizar un *continuum* de acciones preventivas y terapéuticas integrales que sean sensibles a las necesidades de esta población, promoviendo un enfoque eficaz e integral. 

La teoría no solo informa la práctica, sino que también guía la creación de protocolos operativos, diagramas de flujo y otros instrumentos metodológicos que pueden ayudar a la atención de enfermería. Estas herramientas pueden contribuir a la sistematización de la atención, asegurando un enfoque coherente y cualificado en diferentes escenarios sanitarios, destacando su aplicabilidad práctica.

## Data Availability

Los conjuntos de datos relacionados con este artículo estarán disponibles previa solicitud al autor correspondiente.
